# Inhibition of epidermal growth factor receptor attenuates atherosclerosis via decreasing inflammation and oxidative stress

**DOI:** 10.1038/srep45917

**Published:** 2017-04-04

**Authors:** Lintao Wang, Zhouqing Huang, Weijian Huang, Xuemei Chen, Peiren Shan, Peng Zhong, Zia Khan, Jingying Wang, Qilu Fang, Guang Liang, Yi Wang

**Affiliations:** 1 Chemical Biology Research Center, School of Pharmaceutical Sciences, Wenzhou Medical University, Wenzhou, Zhejiang, 325035, China; 2 Department of Cardiology, the First Affiliated Hospital, Wenzhou Medical University, Wenzhou, Zhejiang, 325035, China; 3 Department of Pathology and Laboratory Medicine, Western University, London, ON N6A5C1, Canada

## Abstract

Atherosclerosis is a progressive disease leading to loss of vascular homeostasis and entails fibrosis, macrophage foam cell formation, and smooth muscle cell proliferation. Recent studies have reported that epidermal growth factor receptor (EGFR) is involved vascular pathophysiology and in the regulation of oxidative stress in macrophages. Although, oxidative stress and inflammation play a critical role in the development of atherosclerosis, the underlying mechanisms are complex and not completely understood. In the present study, we have elucidated the role of EGFR in high-fat diet-induced atherosclerosis in apolipoprotein E null mice. We show increased EGFR phosphorylation and activity in atherosclerotic lesion development. EGFR inhibition prevented oxidative stress, macrophage infiltration, induction of pro-inflammatory cytokines, and SMC proliferation within the lesions. We further show that EGFR is activated through toll-like receptor 4. Disruption of toll-like receptor 4 or the EGFR pathway led to reduced inflammatory activity and foam cell formation. These studies provide evidence that EGFR plays a key role on the pathogenesis of atherosclerosis, and suggests that EGFR may be a potential therapeutic target in the prevention of atherosclerosis development.

Coronary atherosclerosis is the principal cause of coronary artery disease and, therefore, a major cause of mortality and morbidity globally^1^,^2^. Atherosclerosis is now recognized as a systemic, lipid-driven inflammatory disease of medium-sized and large arteries leading to multifocal plaque development^3^,^4^,^5^. The formation and progression of atherosclerotic plaques involves aberrant inflammatory cell recruitment, foam cell formation, smooth muscle cell (SMC) proliferation and increased matrix synthesis, production of reactive oxygen species (ROS), and arterial remodeling^6^,^7^. Among these changes, chronic inflammation^8^ and ROS^9^,^10^ appear to play dominant roles. During the inflammatory stage of atherosclerosis, low-density lipoprotein (LDL) is taken up in the arterial wall and is oxidized by excessive ROS. Macrophages scavenge oxidized-LDL (ox-LDL) forming lipid-laden foam cells^11^. Studies have shown that ox-LDL also induces ROS production and release of inflammatory factors, which attribute for the progression of atherosclerosis^12^,^13^. The mechanisms driving ox-LDL-induced inflammation, increased oxidative stress, and atherosclerotic lesion progression are not fully defined.

Epidermal growth factor receptor (EGFR; also known as ErbB1) has recently been implicated in vascular pathophysiological processes associated with excessive remodeling. Activation of EGFR occurs either by binding of ligands such as epidermal growth factor (EGF) and heparin bound-EGF, or by transactivation. EGFR is expressed in macrophages, vascular smooth muscle cells, endothelial cells, and cardiomyocytes, and these cells also secrete EGFR ligands. It has been reported that EGFR plays a role in foam cell transformation, and cellular dysfunction and proliferation of vascular SMCs^14^. EGFR activation by metalloproteinase meprin-α mediates ox-LDL-induced oxidative stress in macrophages^15^. Furthermore, EGFR leads to downstream activation of transcription factors such as nuclear factor-κB (NF-κB) and stimulates pro-inflammatory gene transcription in macrophages^16^,^17^,^18^. Recent findings have also suggested that EGF-like ligands may serve as biomarkers for active inflammatory atherosclerosis in a primate model of atherosclerosis^19^. We have recently shown that inhibition of EGFR effectively protects cardiac damage and remodeling by attenuating oxidative stress in a type 1 diabetic mice model^20^. Taken together, these findings suggest an important role of EGFR in atherosclerosis. Uncovering this role and the mechanism of EGFR activation may lead to the development of new therapeutic modalities for patients with coronary artery disease.

In the present study, we have deciphered the role of EGFR in atherosclerotic lesion formation by utilizing the apolipoprotein E (ApoE) null mice. We inhibited EGFR in these mice by two specific small-molecule EGFR inhibitors AG1478 and 542 (Fig. 1a). We have recently shown that these inhibitors effectively block EGFR activation and attenuate angiotensin II-induced cardiac hypertrophy and dysfunction^21^. We have further delineated a novel mode of EGFR activation in atherosclerosis. Our studies show that EGFR is phosphorylated and activated in atherosclerotic lesion formation. Inhibition of EGFR prevents oxidative stress, induction of inflammatory cytokines, and foam cell formation. We further show that EGFR activation in macrophages involves toll-like receptor 4.

## Results

### Increased EGFR phosphorylation in aortas of HFD-fed ApoE^−/−^ mice

**Figure 1:**
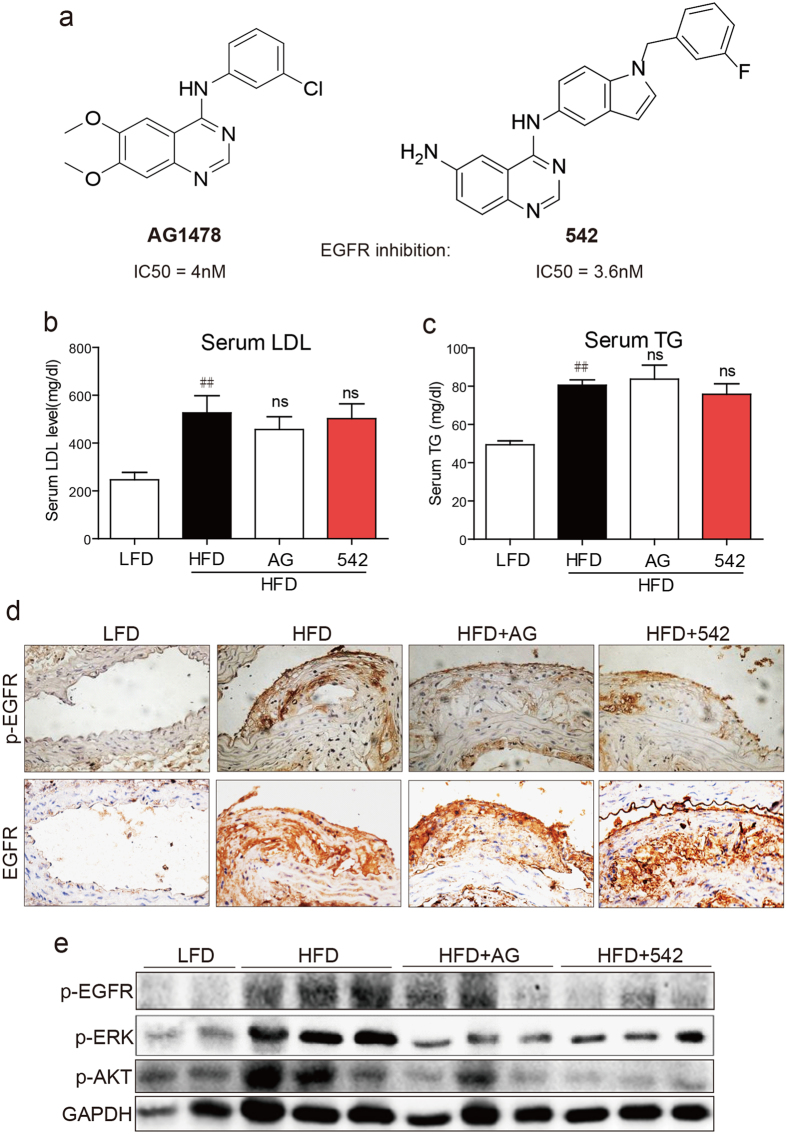
Administration of EGFR inhibitors blocked EGFR signaling activation in HFD-fed ApoE^−/−^ mouse artery. (**a**) The structures of AG1478 and compound 542. ApoE^−/−^ mice were fed with HFD for 8 weeks, and treated with AG1478 (AG, 10 mg/kg/day) or 542 (10 mg/kg/day) for 8 weeks by oral gavage. (**b**,**c**) Serum levels of LDL and TG. (**d**) Representative microscopic images of EGFR and p-EGFR immunochemical staining in artery tissues. (**e**) Western blot analysis of p-EGFR, p-AKT and p-ERK in artery tissues, with the densitometric quantifications shown in Supplementary Fig. S3. The gels were run under the same experimental conditions. Shown are cropped gels/blots (The gels/blots with indicated cropping lines are shown in the Supplementary Fig. 20). (LFD = low fat diet, HFD = high fat diet; n = 7 in each group; ^##^P < 0.01, vs LFD; ns, not significant vs HFD). The quantification results for all staining images were shown in the Supplementary File.

We first wanted to know if inhibiting EGFR alters serum lipid levels since elevated low-density lipoproteins (LDL) have been shown to be strongly related to the development of atherosclerosis. ApoE^−/−^ mice fed a high fat diet (HFD) exhibited increased serum levels of LDL and triglycerides (TG) as compared to mice fed a control/low fat diet (LFD) (Fig. 1b). We also tested the serum insulin level and found that HFD induced the increase in serum insulin while AG or 542 did not affect the insulin level (Supplementary Fig. S1). Inhibiting EGFR through 542 or AG1478 for 8 weeks showed no significant differences in the levels of serum lipids between the HFD mice and the treated groups (Fig. 1b,c). Examination of aorta tissues by immunohistochemistry showed increased levels of EGFR expression and phosphorylation in HFD-fed mice compared to LFD-fed mice (Fig. 1d and Supplementary Fig. S2). Interestingly, treatment of mice with 542 and AG1478 reduced the levels of p-EGFR immunoreactivity but not total EGFR. We also noted activation of predominant signaling proteins downstream of EGFR, namely extracellular signal-regulated kinase (ERK) and Akt. Immunofluorescent staining analysis for p-ERK and p-AKT in aorta tissues found that administration with EGFR inhibitors significantly blocked HFD-induced ERK and Akt phosphorylation in aortas of ApoE^−/−^ mice (Supplementary Fig. S3a–d). Proteins isolated from aorta tissues confirmed these results (Fig. 1e and Supplementary Fig. S4a–d). These results show increased EGFR phosphorylation and activity in atherosclerotic lesions in mice.

Atherogenesis is characterized by developing atheromas driven by progressive uptake of LDL cholesterol by macrophages, becoming lipid-laden foam cells accumulated in the subendothelial space. Additionally, the aberrant growth of SMCs and endothelial cells (ECs) create intimal thickening, and together with foam cells, produce a local environment containing a wide range of secreted mediators such as growth factors and pro-inflammatory molecules. Therefore, all three cell types (macrophages, SMCs, and ECs) contribute to the development of atherosclerosis. We performed the evaluation of p-EGFR localization at the atherosclerotic plaques in aortas of the ApoE^−/−^ mice by colocalization immunofluorescence staining. The results indicated that p-EGFR were increased in these three cell types in HFD mice relative to control mice (Supplementary Fig. S5). However, statistical analysis shows that the ratio of p-EGFR-positive macrophages in total macrophages (41.85%) is higher than the ratio of p-EGFR-positive SMCs in total SMCs (28.84%) and the ratio of p-EGFR-positive ECs in total ECs (7.13%). Thus, our findings suggest that macrophages are mainly associated aberrant EGFR phosphorylation in the lesion. Despite the critical role of SMCs and ECs, we selected macrophages for *in vitro* study.

### AG1478 and 452 treatment prevented atherosclerotic plaque development in HFD-fed ApoE^−/−^ mice

**Figure 2:**
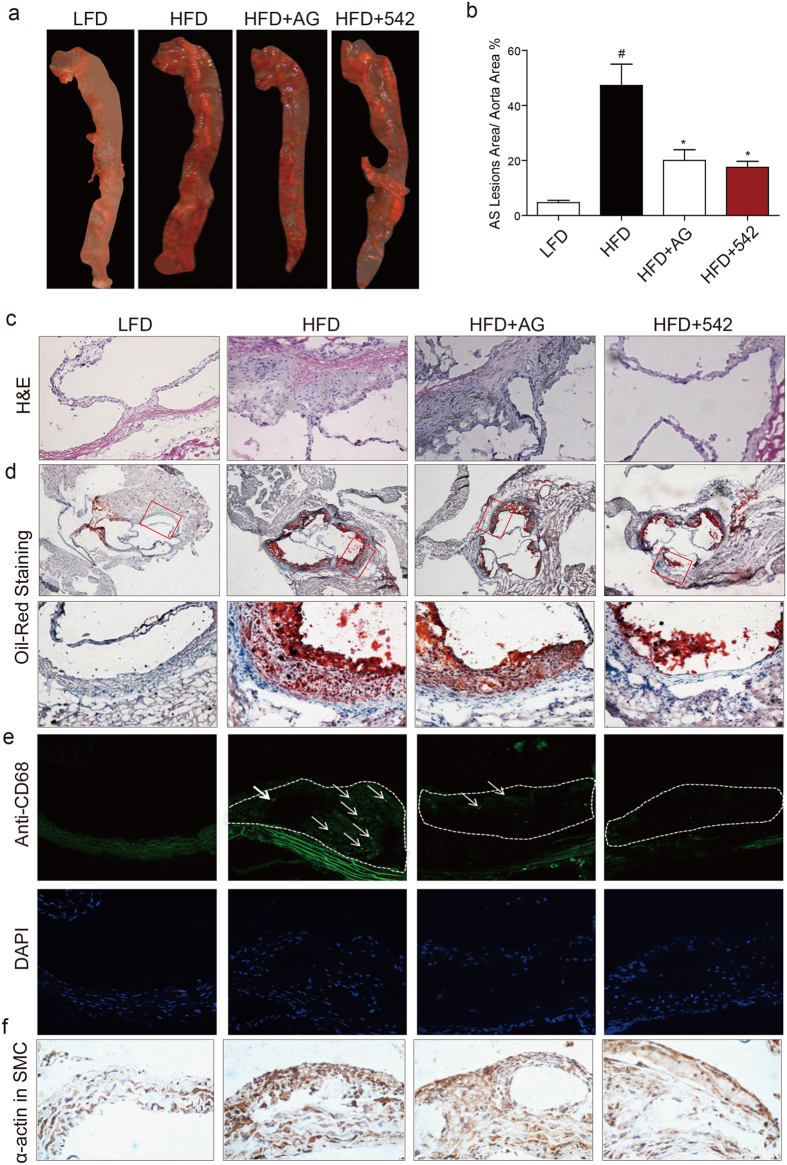
EGFR inhibitors prevented atherosclerotic plaque development in HFD-fed ApoE^−/−^ mice. ApoE^−/−^ mice were fed with HFD for 8 weeks, and treated with AG1478 (AG, 10 mg/kg/day) or 542 (10 mg/kg/day) for 8 weeks by oral gavage. (**a**,**b**) Atherosclerosis plaque staining in the artery using Oil Red staining (**a**), with the quantification of atherosclerotic plaque lesion area (**b**) (n = 7; ^#^P < 0.05, vs LFD; *P < 0.05, vs HFD). (**c**) H&E staining in the aortic valve. (**d**) Oil Red O staining in aortic valve (lower panels show higher magnification). (**e**) Immunofluorescence staining with anti-CD68 in the artery tissues. (**f**) Histochemical staining with anti-α-smooth muscle actin in the artery tissues. All images are representative from 7 mice per group, and the quantification results for all staining images were shown in the Supplementary File.

Although we did not find a difference in serum lipid levels upon EGFR inhibition, reduced EGFR activation in aortas prompted us to examine differences in the degree of atherosclerotic lesions. We performed Oil Red O staining of the entire aorta to measure the severity of these lesions. Our results show significantly increased lesion area in ApoE^−/−^ mice fed a HFD compared to LFD as expected (Fig. 2a,b). Treatment of mice with AG1478 and 542 decreased the atherosclerotic lesion area to approximately half of that observed in untreated HFD-fed mice (Fig. 2a,b). Additional assessment through H&E and Oil Red O staining showed that the plaque areas in the aortic sinus of EGFR inhibitor-treated mice were significantly smaller than in untreated HFD-fed mice (Fig. 2c,d, and Supplementary Fig. S6a). Increased plaque area accompanied increased macrophage infiltration as assessed through CD68 staining (Fig. 2e and Supplementary Fig. S6b). Likewise, smooth muscle proliferation in the aortic sinus of untreated mice were significantly higher than in mice treated with EGFR inhibitors (Fig. 2f, and Supplementary Fig. S6c). All these pathological changes were attenuated by administration with either AG1478 or 542.

A hallmark of a variety of fibrotic diseases, including atherosclerosis, is extensive deposition of extracellular matrix. We tested the effect of EGFR inhibition on fibrosis in aorta tissues of HFD-fed ApoE^−/−^ mice. Treatment of mice with AG and 542 prevented HFD-induced collagen deposition as highlighted by Masson Trichrome and Sirus Red staining (Supplementary Fig. S7a,b). These results were confirmed by determining mRNA levels of collagen 1, and fibrogenic factors connective tissue growth factor (C-TGF) and transforming growth factor-β1 (TGF-β). In addition, we assessed TGF-β protein levels and show that both AG and 542 prevented HFD-induced expression of TGF-β (Supplementary Fig. S7c–f). These results suggest that EGFR inhibition renders ApoE^−/−^ mice resistant to atherosclerosis.

### AG1478 and 542 inhibited HFD-induced inflammation and oxidative stress in aortas

**Figure 3:**
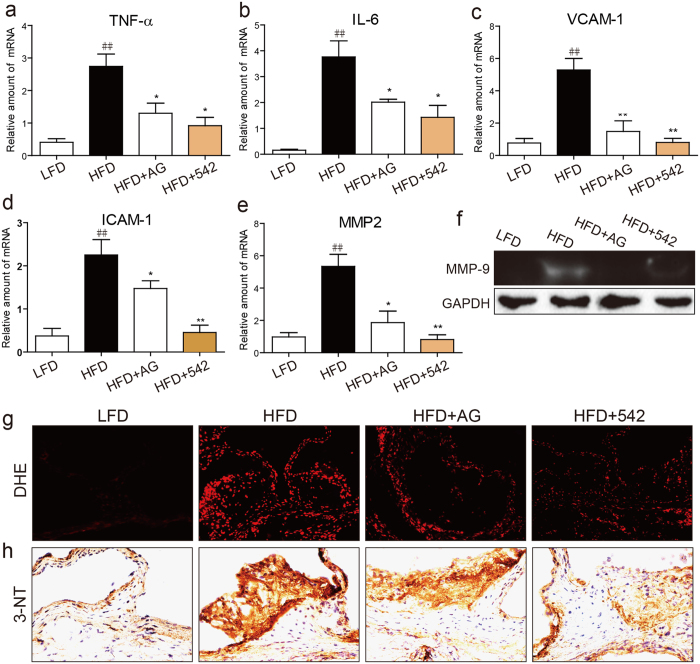
EGFR inhibitors prevented inflammation and oxidative stress in the atherosclerotic plaques of HFD-fed ApoE^−/−^ mice. ApoE^−/−^ mice were fed with HFD for 8 weeks, and treated with AG1478 (AG, 10 mg/kg/day) or 542 (10 mg/kg/day) for 8 weeks by oral gavage. (**a**–**e**) Real time qPCR analysis of TNF-α (**a**), IL-6 (**b**), VCAM-1 (**c**), ICAM-1 (**d**), MMP2 (e). (**f**) MMP-9 activity in the atherosclerotic plaques as measured by gelatin zymography. The gels were run under the same experimental conditions. Shown are cropped gels/blots (The gels/blots with indicated cropping lines are shown in the Supplementary Fig. 20). (n = 7 per group, ^#^P < 0.05, vs LFD; *P < 0.05, **P < 0.01, vs HFD). (**g**,**h**) Representative images of Dihydroethidium (DHE) and anti-3-Nitrotyrosine (3-NT) staining in aortic valve tissues. The quantification results for all staining images were shown in the Supplementary File.

We sought to clarify whether inflammation and oxidative stress were involved in the attenuation of atherosclerotic plaque development by EGFR inhibition. The levels of inflammatory factors and adhesion molecules including tumor necrosis factor-α (TNF-α), interleukin-6 (IL-6), vascular cell adhesion molecule-1 (VCAM-1), and intracellular adhesion molecule-1 (ICAM-1) in atherosclerotic aortas were markedly higher in HFD-fed mice compared to LFD-fed control mice and EGFR inhibitor-treated mice (Fig. 3a–d). The fact that EGFR inhibition prevented induction of inflammatory cytokines and adhesion molecules point to an important role of EGFR early in the disease course.

We next examined parameters of tissue remodeling as excessive inflammatory cytokines may increase the expression and activity of matrix metalloproteinases (MMPs). As shown in Fig. 3e,f, MMP2 expression and MMP9 activity in aortas of HFD-fed mice were significantly increased compared to the LFD-fed mice. Both MMP2 and MMP9 have been shown to be involved in atherosclerosis^22^ and serve as markers of tissue remodeling and progression of atherosclerotic lesions. Treatment of mice with 452 and AG1478 reduced MMP2 and MMP9 to levels comparable to LFD-fed mice. Mirroring the pattern of inflammatory markers, dihydroethidium (DHE) fluorescence staining for reactive oxygen species (ROS) and nitrotyrosine (3-NT) immunohistochemistry showed increased oxidative stress in aortas of HFD-fed mice (Fig. 3g,h, and Supplementary Fig. S8a,b). Both measures of oxidative stress were decreased by AG1478 and 542, indicating that EGFR inhibition reduces inflammation and ROS in the development of atherosclerosis.

### EGFR inhibitors suppress inflammation in ox-LDL-stimulated macrophages and vascular smooth muscle cells (SMCs)

**Figure 4:**
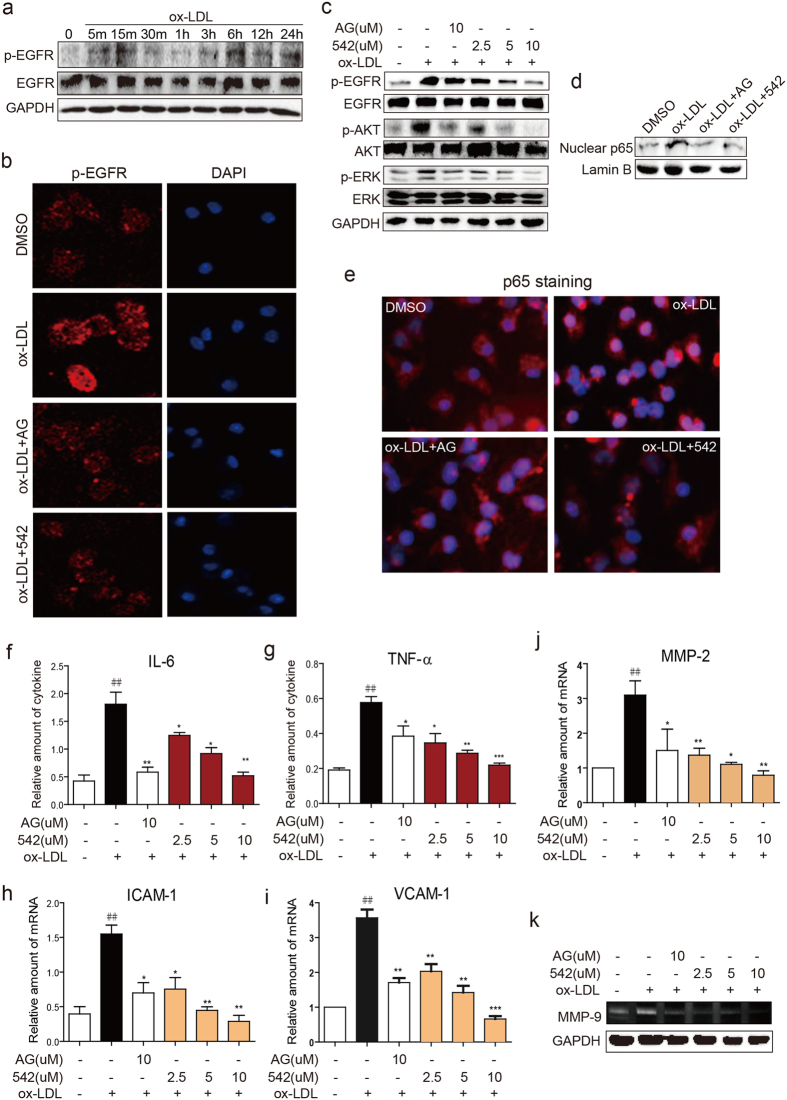
Inhibiting EGFR blocks ox-LDL-induced inflammation in macrophages. (**a**) ox-LDL activates EGFR in macrophages. MPMs were stimulated with ox-LDL (50 μg/mL) for different time points. Cell lysates were analyzed for p-EGFR and EGFR. (**b**,**c**) AG and 542 suppressed ox-LDL-induced activation of EGFR. MPMs were pretreated with 542 (10 μM or indicated concentrations), AG1478 (10 μM), or vehicle (DMSO, 1 μL) for 1 h and then stimulated with ox-LDL (50 μg/mL) for 15 min. Immunofluorescence staining for p-EGFR and DAPI was performed (**b**) and the levels of p-EGFR, p-ERK, and p-AKT in cell lysates were detected by western blot (**c**). (**d**,**e**) AG and 542 suppressed ox-LDL-induced activation of NF-κB. MPMs were pretreated with 542 (10 μM), AG1478 (10 μM), or vehicle (DMSO, 1 μL) for 1 h and then stimulated with ox-LDL (50 μg/mL) for 1 h. Levels of nuclear NF-κB p65 were assessed by western blotting with Lamin B as a loading control (**d**), or were detected by anti-p65 immunofluorescence staining (**e**). (**f**–**j**) AG and 542 inhibited ox-LDL-induced release of cytokines. MPMs were pretreated with 542 (2.5, 5 or 10 μM) and AG1478 (10 μM) for 1 h and then stimulated with ox-LDL (50 μg/mL) for 24 h (in panels f and g) or 6 h (in panels h–j). The levels of IL-6 (**d**) and TNF-α (**e**) in the cultural medium were detected by ELISA. The mRNA levels of ICAM-1 (**h**), VCAM-1 (**i**), and MMP-2 (**j**) were detected by real-time qPCR assay. (**k**) AG and 542 inhibited ox-LDL-induced MMP9 activity. MPMs were pretreated with 542 or AG1478 at indicated concentrations for 1 h and then stimulated with ox-LDL (50 μg/mL) for 48 h. MMP-9 activity in the medium was measured by gelatin zymography. (n = 4 independent experiments, ^##^P < 0.01, vs control; *P < 0.05, **P < 0.01, ***P < 0.001, vs ox-LDL). For panels a, c, d, and k, the gels were run under the same experimental conditions. Shown are cropped gels/blots (The gels/blots with indicated cropping lines are shown in the Supplementary Fig. 20). The quantification results for all staining images were shown in the Supplementary File.

EGFR signaling has been shown to mediate lipopolysaccharide-induced inflammation via regulating the activation of nuclear factor-κB (NF-κB) in macrophages^18^. Given that EGFR inhibitors 452 and AG1478 are able to attenuate atherosclerosis through reducing inflammation in mice, we investigated the anti-inflammatory effects of EGFR inhibitors in oxidized-LDL (ox-LDL)-stimulated primary macrophages. Brief exposure of macrophages to ox-LDL induced EGFR phosphorylation, but not EGFR expression, as detected by western bott method (Fig. 4a and Supplementary Fig. S9) and immunofluorescence staining (Fig. 4b and Supplementary Fig. S10). The levels of p-EGFR were greatly reduced when cells were pre-treatment with 542 or AG1478 (Fig. 4b and Supplementary Fig. S10). Downstream signaling proteins Akt and ERK were also phosphorylated by ox-LDL and inhibited with 542 and AG1478 (Fig. 4c and Supplementary Fig. S11a). As macrophage NF-κB^18^ has been shown to be critical in inflammation, we assessed its activation by western blotting and cell staining. Our results show increased NF-κB p65 subunit in the nuclear protein fraction and increased nuclear staining of macrophages stimulated by ox-LDL, as detected by western bott method (Fig. 4d and Supplementary Fig. S11b) and immunofluorescence staining (Fig. 4e and Supplementary Fig. S12). In both assays, 542 and AG1478 markedly inhibited ox-LDL-induced NF-κB activation.

Activation of EGFR and downstream signaling proteins by ox-LDL was also associated with induction of pro-inflammatory cytokines TNF-α and IL-6 at both protein and mRNA levels in cultured macrophages. As expected, AG1478 or 542 prevented this induction (Fig. 4f,g, Supplementary Fig. S13a,b). mRNA analysis also showed that AG1478 and 542 suppressed the expression of adhesion molecules ICAM-1 and VCAM-1 induced by ox-LDL (Fig. 4h,i). We then examined MMPs as our studies in aorta tissues showed dysregulated expression and activity in atherosclerotic lesions. In cultured macrophages, ox-LDL increased MMP2 expression and MMP9 activity and both of these changes were prevented by AG and 542 pretreatment (Fig. 4j,k). We also tested the anti-inflammatory effects of EGFR inhibitors in cultured SMCs and show responses similar to macrophages (Supplementary Fig. S14a,b).

### EGFR inhibition prevented ox-LDL-induced ROS production, mitochondrial damage, and foam cell formation

**Figure 5:**
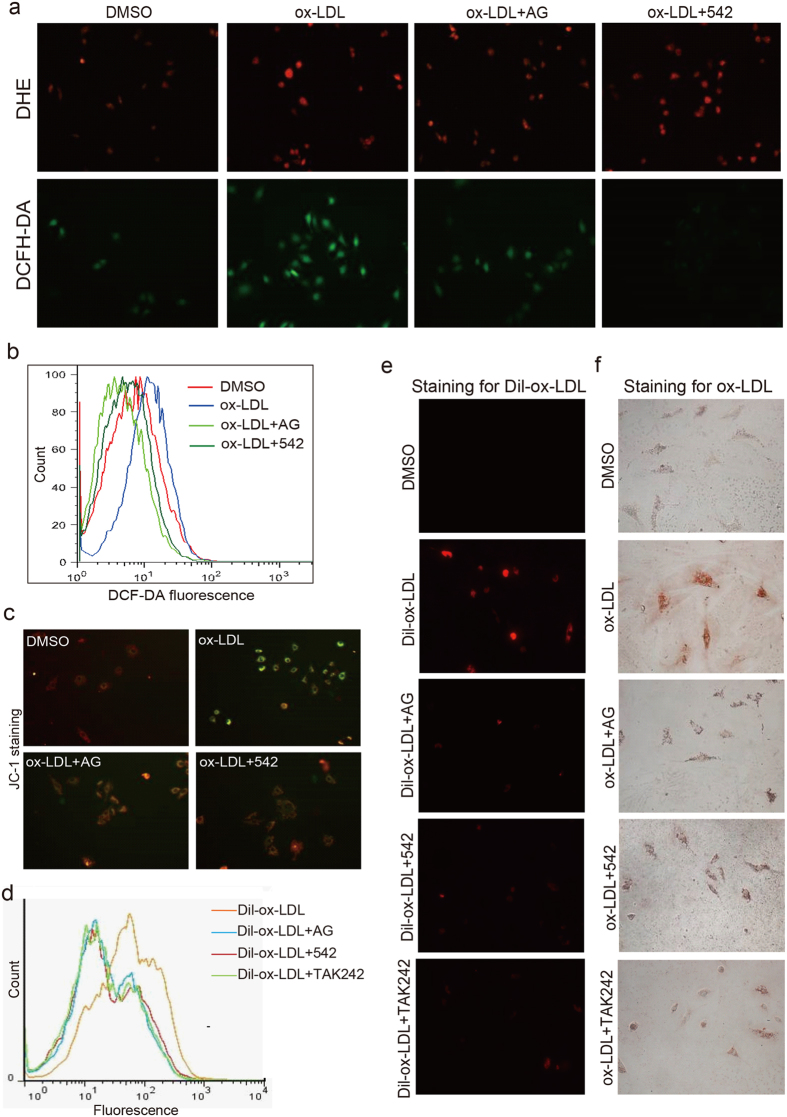
AG and 542 inhibit ox-LDL-induced ROS production and foam cell formation in primary macrophages. (**a**,**b**) AG and 542 inhibited the production of O_2_^-^ or H_2_O_2_ by ox-LDL. Primary macrophages were pretreated with 542 and AG at 10 μM for 1 h, followed by the incubation with ox-LDL (50 μg/mL) for 30 min. DHE and DCFH-DA probes were loaded and cells were detected using fluorescence microscope (**a**). DCFH-DA probes were loaded and cells were analyzed by flow cytometry for H2O2 level (**b**). (**c**) AG and 542 attenuates ox-LDL-induced mitochondrial injury. Primary macrophages pretreated with AG or 542 at 10 μM for 1 h were incubated with oxLDL (50 μg/mL) for 24 h. Cells were subjected to JC-1 staining for mitochondrial membrane potential analysis. (**d**,**e**) Primary macrophages were pretreated with 542, AG1478, or TAK242 at 10 μM for 1 h, followed by the incubation with Dil-ox-LDL (100 μg/mL) for 30 min. Cells were then processed by flow cytometry (**d**) or fluorescence imaging (**e**). (**f**) Primary macrophages were pretreated with 542, AG1478, or TAK242 at 10 μM for 1 h and then stimulated with ox-LDL (100 μg/mL) for 30 min and then stained with Oil Red O. (Data are representative from n = 4 independent experiments; the quantifications were shown in the Supplementary file). The quantification results for all staining images were shown in the Supplementary File.

Increased EGFR phosphorylation was found to play a vital role in the production of ROS by ox-LDL in macrophages^15^. Here, we determined the effects of EGFR inhibitors on ox-LDL-stimulated ROS generation in macrophages. Exposure of macrophages to ox-LDL for 6 h significantly increased ROS generation as indicated by DCFH-DA/DHE fluorescence staining (Fig. 5a and Supplementary Fig. S15) and flow cytometry (Fig. 5b). Pretreatment with 452 or AG1478 was able to block increased ROS generation. Similar results were obtained in SMCs (Supplementary Fig. S16). To understand how EGFR induces ROS production following ox-LDL stimulation, we tested the effects of EGFR inhibitors on the expression and activity of NADPH oxidase (NOX) in macrophages. NOX1 has recently been shown to activate infiltrating immune cells, increasing ROS levels in aortic sinus of diabetic mice^23^. Our results showed that both AG1478 and 451 significantly reversed ox-LDL-induced NADP/NADPH ratio and inhibited ox-LDL-induced NOX-1 expression (Supplementary Fig. S17). In addition, we tested the determination of NO level and iNOS expression in oxLDL-stimulated macrophages. It was observed that pre-treatment with EGFR inhibitors significantly blocked oxLDL-induced overproduction of NO and overexpression of iNOS (Supplementary Fig. S18).

We next examined mitochondrial membrane potential as it is well known that increased ROS levels result in the mitochondrial dysfunction. Loss of mitochondrial membrane potential (Dψm) is catastrophic for cells and leads to the release of cytochrome C into the cytosol. We tested mitochondrial membrane potential loss by using potential-sensitive ratiometric fluorescence dye JC-1. As shown in Fig. 5c, ox-LDL caused a pronounced decrease in mitochondrial Dψm indicating a reduction of highly energized mitochondria. In contrast, pretreatment with EGFR inhibitors (AG1478 or 542) for 1 h attenuated the ox-LDL-induced decrease in mitochondrial Dψm.

Once lipids are taken up in the arterial wall and oxidized by ROS, macrophages scavenge these modified lipids and become foam cells. We, therefore, investigated the role of EGFR in ox-LDL uptake by macrophages. Cells were incubated with DiI-labled ox-LDL (DiI-ox-LDL) with or without pretreatment with EGFR inhibitors and analyzed by fluorescence microscopy and flow cytometry. Here, we report that inhibition of EGFR prevented ox-LDL update in macrophages (Fig. 5d,e, and Supplementary Fig. S19a). We confirmed these results by staining macrophages exposed to ox-LDL with Oil Red O (Fig. 5f and Supplementary Fig. S19b). These studies show that EGFR inhibition reduced formation of foam cells.

### ox-LDL induces EGFR activation through toll-like receptor/Src in macrophages

**Figure 6:**
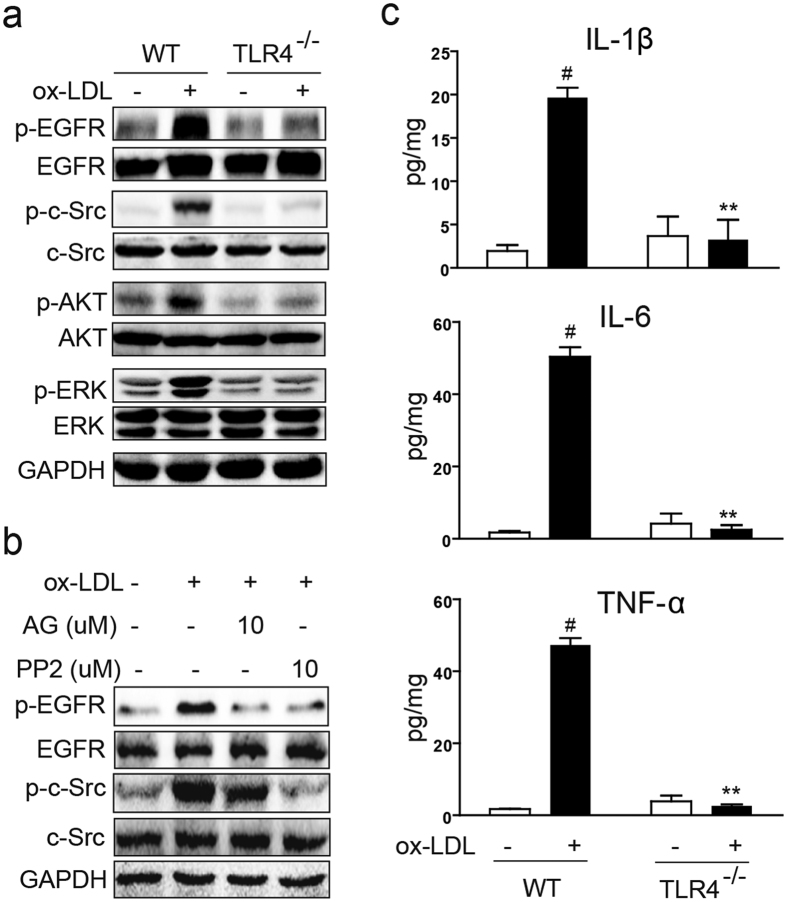
oxLDL-induced EGFR activation requires TLR4/c-Src. (**a**) EGFR is not activated by ox-LDL in the TLR4 knockout-derived macrophages. Primary macrophages isolated from TLR4 knockout mice and C57/B6 WT mice were stimulated with ox-LDL (50 μg/mL) for 15 min. p-EGFR/EGFR, p-c-Src/c-Src, p-AKT/AKT, and p-ERK/ERK levels were determined by western blotting. (**b**) c-Src inhibitor PP2 prevents ox-LDL-induced EGFR activation. Primary macrophages were pretreated with AG1478 or PP2 at 10 μM for 1 h, followed by the incubation with ox-LDL (50 μg/mL) for 15 min. Total proteins were extracted to detect the levels of p-EGFR/EGFR and p-c-Src/c-Src using western blot analysis. (**c**) Primary macrophages isolated from TLR4 knockout mice and C57/B6 and stimulated with ox-LDL (50 μg/mL) for 24 h. Culture medium was used to detect the levels of TNF-α, IL-6 and IL-1β by ELISA. (n = 4 independent experiments, ^#^P < 0.05, vs control WT; **P < 0.01, vs ox-LDL-WT). For panels a and b, the gels were run under the same experimental conditions. Shown are cropped gels/blots (The gels/blots with indicated cropping lines are shown in the Supplementary Fig. 20).

Our studies have shown that EGFR inhibition prevented atherosclerotic lesion formation, inflammation, ROS generation, and foam cell formation. However, it remains unclear as to how ox-LDL activates EGFR signaling. EGFR ligands including heparin binding-EGF (HB-EGF)^24^,^25^, epiregulin (EREG)^26^, TGF-α^25^,^27^, and β-cellulin^28^ are associated with human atherosclerosis and potentially may contribute to the EGFR activation. Our studies show rapid phosphorylation of EGFR suggesting direct activation rather than through elaboration of typical EGF ligands. Recent studies have suggested that EGFR can also be activated without the typical ligands^29^, and it can function in intracellular membranes^30^. Toll-like receptor 4 (TLR4) has been reported to be directly activated by ox-LDL and mediate pathological pathways and phenotypes^31^,^32^,^33^. In addition, expression of TLR4-induced genes in lipopolysaccharide-stimulated myeloid cells requires EGFR kinase activity^18^. We have also found that TLR4 and c-Src mediate palmitic acid-induced EGFR activation in cardiomyocyte-like H9c2 cells^34^. Thus, we tested whether TLR4/c-Src mediates ox-LDL-induced EGFR activation in macrophages. We collected primary macrophages from TLR4^−/−^ mice and wildtype (WT) mice. Protein analysis of cultured primary macrophages showed that ox-LDL increased c-Src and EGFR/ERK/AKT phosphorylation in WT macrophages but not in macrophages derived from TLR4^−/−^ mice (Fig. 6a). In addition, pretreatment of primary macrophages with AG1478 and c-Src inhibitor (PP2) reduced EGFR phosphorylation (Fig. 6b). Interestingly, AG1478 pretreatment only blocked EGFR phosphorylation but did not alter c-Src possibly indicating that c-Src is upstream of EGFR activation.

We reasoned that if TLR4 mediated ox-LDL-induced EGFR phosphorylation then inflammatory activity downstream of EGFR would not be evident in cells from TLR^−/−^ mice. Indeed, ox-LDL failed to induce IL-1β, IL-6, and TNF-α release from TLR4^−/−^ macrophages (Fig. 6c). Similarly, inhibition of TLR4 through TAK242 prevented ox-LDL update and foam cell formation (Fig. 5d–f). These results suggest that TLR4/c-Src signaling mediates EGFR activation downstream of ox-LDL and leads to foam cell formation.

## Discussion

The development of atherosclerosis is tightly associated with chronic inflammation and oxidative stress in the arterial plaque^3^,^4^,^35^. Fibro-fatty plaque formation and SMC proliferation are also hallmarks of atherosclerosis. In the present study, we evaluated whether EGFR-dependent pathways play a role in the development of atherosclerosis in ApoE^−/−^ mice. Mice fed a HFD for 8 weeks showed accelerated atherosclerotic lesions characterized by accumulation of SMCs and macrophages. In addition, formation of foam cells, induction of inflammatory factors including IL-6, ICAM-1 and TNF-α, accompanied increased EGFR phosphorylation and activity. Inhibition of EGFR using AG1478 or compound 452 significantly ameliorated these abnormalities without altering serum LDL levels. Our results indicated that p-EGFR were increased in all three cell types (macrophages, SMCs, and ECs), which contribute mainly to atherosclerosis, in HFD-fed ApoE^−/−^ mice. We confirmed our findings in cultured macrophages and SMCs challenged with ox-LDL. Finally, we identified a novel mechanism of oxLDL-induced EGFR activation involving TLR4 in macrophages. These findings indicate a detrimental effect of activated EGFR in the pathogenesis of atherosclerosis, and that exacerbated EGFR phosphorylation contributes to the progression of atherosclerotic plaque formation, likely through increased inflammation and oxidative stress.

Oxidative stress plays a key role in the progression of cardiovascular disease. In particular, ROS very commonly accompanies the development of typical characteristics of atherosclerosis^10^,^36^. Excessive ROS generation can directly damage the cell membrane, proteins and DNA. Mitochondrial DNA has also been proposed to be susceptible to oxidative damage^37^,^38^. Recent studies show that increasing ROS production participates in inflammation, disturbed blood blow and abnormal shear stress, and arterial wall remodeling^39^,^40^. In addition, Park and colleagues^7^ reported that oxidative stress contributes to structural remodeling through SMC proliferation and enhanced inflammation. In the present work, we found oxidative stress markers in the arteries of ApoE^−/−^ mice were increased. Increased oxidative damage was associated with artery remodeling and enhanced inflammation *in vivo* (Figs 3 and 4). Interestingly, oxidative stress as well as SMC proliferation was significantly attenuated by AG1478 and 452 treatments. These results confirm that ROS is involved in the development of atherosclerosis and clearly show the involvement of EGFR in ROS production and SMCs proliferation. The mechanisms leading to enhanced ROS generation through EGFR are just recently being clarified and may involve EGFR/AKT^41^,^42^. MAPK pathways are also reported to be involved in the ROS production in macrophages^43^. In addition, several reports confirmed EGFR-PI3K-AKT/ERK signaling pathway responsible for ROS generation^44^,^45^,^46^. In a related system, we have shown that EGFR inhibitors significantly blocked NOX expression and activity in high glucose-induced H9c2 cell^20^. Here, we show that the same EGFR/AKT-ERK activation pathway enhances ROS production in atherosclerotic lesion of ApoE^−/−^ mice, which were markedly reversed by EGFR inhibitors AG1478 or 452. We also show that ox-LDL-stimulated macrophages utilize the NOX and iNOS pathways for ROS generation.

In addition to ROS (and likely downstream of ROS), inflammation plays an important role in the initiation and progression of atherosclerosis^8^,^47^. Multiple cell types including monocytes/macrophages, T-lymphocytes, SMCs and mast cells^8^ are present in atherosclerotic plaques from the earliest lesions to ruptured plaques. These cells accompany various inflammatory and tissue remodeling factors including TNF-α, IL-6, ICAM-1, VCAM-1 and MMPs^48^. We established that increased EGFR signaling activation is associated with artery inflammation and lipid accumulation in macrophages. Recently, we have found that administration of EGFR inhibitors (AG1478 and 542) significantly prevented HFD-induced inflammation in ApoE^−/−^ mouse hearts^34^ and both ApoE−/− and C57B/L6 mouse kidneys^49^. That is to say, EGFR inhibition may prevents systemic inflammatory changes in HFD-fed mice. EGFR inhibition using AG 1478 or 452 alleviated atherosclerotic lesions in ApoE^−/−^ mice through decreasing macrophages infiltration, foam cell formation and possibly matrix metalloproteinase secretion. These findings suggest EGFR activation is responsible for the pathophysiological development of atherosclerosis. Consistent with our observations, a recent study by Liang *et al*. showed that meprin-α activated EGFR activity to induce oxidative stress in ox-LDL-stimulated macrophage^15^. The authors showed that meprin-α promotes the formation of atherosclerotic plaques and ROS production, and both are reversed with AG1478 treatment. Herein, we observed that all these abnormalities appeared to be reduced by EGFR inhibitor AG1478 or 452, indicating EGFR activation play a critical role.

Our studies have shown that AG1478 and 452 inhibit the phosphorylation of ERK and p65 nuclear translocation in ox-LDL-induced macrophages. This indicates that EGFR functions upstream of ERK and NF-κB in macrophages. Rapid activation of EGFR in cultured cells points to a mode of action rather than elaboration of typical EGF ligands. Rapid activation of EGFR by ox-LDL has been reported in vascular cells^50^,^51^,^52^,^53^, though the mechanisms are unknown. We identified TLR4 as a potential activator of EGFR in macrophages. TLR has been shown to be important in activated macrophages, regulating nucleotide-binding domain and leucine-rich repeat containing (NLR) family, pyrin domain containing 3 (NLRP3) inflammasomes^15^,^54^,^55^,^56^. We showed phosphorylation of EGFR/AKT/ERK to be deficient in macrophages derived from TLR4^−/−^ mice. Moreover, downstream effects of EGFR activation including induction of inflammatory factors (IL-6, IL-1β and TNF-α) and MCP-1 secretion was lacking in TLR4^−/−^ macrophages challenged with ox-LDL. Furthermore, inhibition of TLR4 prevents foam cell formation. These observations suggest that TLR4 plays its pro- activity through regulating the activation of EGFR/AKT/ERK signal pathway.

It is worth noting that many pharmacological interventions, including statins, angiotensin-converting enzyme inhibitors, niacin and calcium channel blockers, target ROS and inflammation to abrogate the development of atherosclerosis^10^. In the present study, the newly synthesized EGFR inhibitor 452 showed effective prevention of atherosclerosis development. The effect produced by 452 was comparable to AG1478 in improving inflammation and ROS production both *in vitro* and *in vivo*. EGFR inhibitors already constitute the first-line therapy for a number of cancers and our studies suggest another clinically significant indication where EGFR inhibitors may be of therapeutic benefit.

## Material and Methods

### Reagents and cell culture

AG1478 were purchased from Sigma-Aldrich (St. Louis, MO). Compound 542 (Fig. 1a) was prepared with a purity of 99.2% as described in our previous study^34^. AG1478 and compound 542 were dissolved in dimethyl sulfoxide (DMSO) for *in vitro* experiments and in 1% sodium carboxyl methyl cellulose (CMC-Na) for *in vivo* experiments. Antibodies against GAPDH, p-EGFR and p-AKT were purchased from Cell Signaling (Danvers, MA, USA). Antibodies against p-ERK, TGF-β, Collagen4, cleaved caspase 3, Bax, Bcl-2, and TLR4 were purchased from Santa Cruz Biotechnology (Santa Cruz, CA). Antibody against CD68 was purchased from Abcam (Cambridge, MA). Human vascular smooth muscle cell line was purchased from R&S Biotech. Co., LTD (Shanghai, China).

### Preparation of mouse peritoneal macrophages

Mouse primary peritoneal macrophages (MPMs) were isolated from C57BL/6 mice and cultured as shown by us previously^57^. Briefly, C57BL/6 mice were simulated by intraperitoneal injection of 6% thioglycollate solution (0.3 g beef extract, 1 g tryptone, 0.5 g sodium chloride dissolved in 100 ml ddH_2_O, and filtrated through 0.22-μm filter membrane, 3 ml per mouse) and kept in a pathogen-free condition for 3 days before mouse peritoneal macrophages (MPMs) isolation. Mice were euthanized by rising CO_2_ inhalation, in accordance with Schedule 1 of the Animals (Scientific Procedures) Act (1986). Total MPMs were harvested by washing the peritoneal cavity with PBS containing 30 mM of EDTA (8 ml per mouse), centrifuged, and suspended in RPMI-1640 medium (Gibco/BRL life Technologies, Eggenstein, Germany) with 10% fetal bovine serum (Hyclone, Logan, UT, USA), 100 U/ml penicillin, and 100 mg/ml streptomycin. Nonadherent cells were removed by washing with medium 3 h after seeding. Experiments were undertaken after the cells were firmly adhered to the culture plates.

### Real-time quantitative PCR

Total RNA was isolated from cells and artery tissues using TRIZOL (Thermo Fisher, Carlsbad, CA). Both reverse transcription and quantitative PCR were carried out using a two-step M-MLV Platinum SYBR Green qPCR SuperMix-UDG kit (Thermo Fisher) in Eppendorf Mastercycler ep realplex detection system (Eppendorf, Hamburg, Germany). Primers were obtained from Thermo Fisher (Shanghai, China). Primer sequences are listed in Supplementary Table S1. mRNA levels of target genes was normalized to β-actin.

### Western immunoblot analysis

Lysates from cells or homogenized artery tissues were separated by 10% SDS-PAGE and electro-transferred onto a nitrocellulose membrane. Each membrane was pre-incubated for 1.5 h at room temperature in Tris-buffered saline (pH 7.6, containing 0.05% Tween 20 and 5% non-fat milk). Membranes were then incubated with specific antibodies. Immunoreactive bands were detected by incubating with secondary antibody conjugated to horseradish peroxidase and visualizing using enhanced chemiluminescence reagent (Bio-Rad, Hercules, CA). The amounts were analyzed using Image J analysis software version 1.38e (NIH) and normalized to their respective controls.

### Oil red staining

Macrophages were incubated with 100 μg/mL ox-LDL (Biomedical Technologies) in RPMI 1640 media for 24 h. At the time of analysis, cells were fixed in 4% paraformaldehyde for 15 min, washed with PBS, and incubated with a 0.5% working solution of Oil Red O (Jiancheng Bioengineering Institute, Nanjing, China) for 15 min.

### MMP-9 gelatinase activity

Following treatment of cell, 25 μL cell-free condition media was collected by centrifugation. Media was mixed with 25 μL of Laemmli buffer without β-mercaptoethanol and separated using 10% SDS-PAGE containing 1 mg/mL gelatin. The gels were incubated in Zymogram renaturing Buffer (0.25% Triton X 100 solution) for 1 h at room temperature followed by incubation overnight in Zymogram developing buffer (50 mmol/L Tris base, 50 mmol/L Tris-HCl, 0.2 mmol/L NaCl, 5 mmol/L CaCl2 and 0.02% Brij 35). Gels were stained with Coomassie Blue R-250 solution to get clear bands against a dark blue background where the proteases had digested the substrate.

### Dil-ox-LDL uptake and binding assays

ox-LDL lipoproteins were labeled with the fluorescent probe DiI. For uptake assays, mouse peritoneal macrophages were incubated in fresh media containing 50 μg/mL DiI-Ox-LDL for 3 h at 37 °C. For the binding assays, cells were incubated for 15 min at 4 °C to stop membrane internalization. Cells were visualized under a Nikon epi-fluorescence microscope equipped with a digital camera (Tokyo, Japan). Finally, cells were analyzed by flow cytometry (FACScalibur; Becton Dickinson, San Diego, CA, USA). The results are expressed in terms of specific median intensity of fluorescence after subtracting auto-fluorescence of cells (absence of DiI-Ox-LDL).

### Enzyme-linked immunosorbent assay

Mouse macrophages were pretreated with the compounds for 2 h, then treated with 50 mg/mL ox-LDL for 24 h. After treatment, the culture media and cells were collected separately. The levels of tumor necrosis factor alpha (TNF-α) and interleukin-6 (IL-6) in the media were determined by enzyme-linked immunosorbent assay (ELISA) (eBioScience, San Diego, CA). The total quantity of the inflammatory factor in the media was standardized to the total protein amount of the viable cell pellets.

### Mitochondrial Membrane Potential (Δ ψ) analysis

Cells were seeded onto glass slides (Orange Scientific. E.U). JC-1 assay reagent (Beyotime BioTech., Nanjing, China) was diluted in culture media and cells were incubated for 20 min to stain the mitochondria. After 2 to 3 rinses, cells were inspected using an Axiovert 200 fluorescent inverted microscope (Zeiss, Germany). Both monomeric (excitation at 488 nm, emission 500–550 nm) as well as aggregation (excitation 488 nm, emission at 575–620 nm) were registered using the microscope.

### Measurements of the level of serum lipid and biochemical indicators

The components of serum lipid including the total triglycerides (TG), low-density lipoprotein (LDL), Total cholesterol (TCH). (Nanjing Jiancheng, Jiangsu, China).

### Determination of ROS generation by fluorescent microscope and flow cytometry

In order to analyze ROS generation, we used Dichloro-dihydro-fluorescein diacetate (DCFH-DA) which measures H_2_O_2_ and allows for ROS determination in live cells. The fluorescence intensity for 10,000 events was acquired using FACS, and cellular images were captured under the Nikon fluorescence microscope.

### Determination of NADPH oxidase activity

After treatments, NADPH oxidase activity in cells was measured using NADP/NADPH Quantification colorimetric Kit (BioVision Inc., Milpitas, CA) as previously described^45^.

### Animal experiments

Male ApoE^−/−^ mice (18–20 g, 8 weeks) on C57BL/6 background were purchased from HFK Bioscience Co. Ltd (Beijing, China). Mice were housed at a constant room temperature with a 12:12 h light–dark cycle and fed with a standard rodent diet. Mice were acclimatized to the laboratory for at least 3 days before initiating studies. All animal care and experimental procedures were approved by the Wenzhou Medical University Animal Policy and Welfare Committee (wydw2014-0058). All animal experiments were performed conform the NIH guidelines (Guide for the care and use of laboratory animals).

ApoE^−/−^ mice were randomly divided into four weight-matched groups (n = 7, total 28 mice). 7 mice were fed with standard animal low-fat diet containing 10 kcal.% fat, 20 kcal.% protein and 70 kcal.% carbohydrate (MediScience Diets Co. LTD, Yangzhou, China, Cat. #MD12031) served as the normal control group (LFD), while the remaining 21 mice were fed with high-fat diet containing 60 kcal.% fat, 20 kcal.% protein and 20 kcal.% carbohydrate (HFD, MediScience Diets Co. LTD, Yangzhou, China, Cat. #MD12033) for 16 weeks. Since 9^th^ week HFD-fed mice were then divided into three groups: HFD (n = 7), AG1478-treated HFD (HFD + AG, n = 7) and 542-treated HFD (HFD+542, n = 7). AG and 542 compounds were administered orally at 10 mg/kg/day for the last 8 weeks. The HFD and LFD groups received 1% CMC-Na solution alone. Bodyweight was recorded weekly after AG/542 administration. Mice were euthanized by rising CO_2_ inhalation, in accordance with Schedule 1 of the Animals (Scientific Procedures) Act (1986), and blood was collected by cardiac puncture into a syringe containing 4% trisodium citrate (1:10, v/v). Artery tissues were embedded in 4% paraformaldehyde for microscopic analysis and/or snap-frozen in liquid nitrogen for gene and protein expression analysis.

### Histology and analysis of atherosclerotic lesions

For analysis of plaque lesion in aortic sinus, the heart and proximal aorta were removed and embedded in optimum cutting temperature compound. Serial 10 μm-thick cryosections from the middle portion of the ventricle to the aortic arch were collected. Sections were stained with oil red O and hematoxylin. For en face analyses of lesions in the entire aorta, whole aorta was dissected out, opened longitudinally from heart to the iliac arteries, and stained with Oil Red O.

Five μm frozen sections were stained with hematoxylin and eosin (H&E) for histopathological observation. Paraffin sections (5 μm) were stained with 0.1% Sirius Red and Masson trichrome for collagen deposition and fibrosis.

### Immunohistochemistry

Paraffin sections were deparaffinization and rehydration. Slides were incubated with 3% H_2_O_2_ for 10 min to block endogenous peroxidase activity. Slides were blocked with 1% bovine serum albumin in for 30 min and then incubated overnight at 4 °C with p-EGFR and smooth muscle α-actin antibody (1:200). Horseradish peroxidase-conjugated secondary antibody (Santa Cruz; 1:500) and DAB were used for detection.

Frozen sections were used for immunofluorescence. Slides were blocked using 1% bovine serum albumin for 30 min and incubated overnight at 4 °C with CD68 antibody (1:200). FITC-conjugated secondary antibody (Santa Cruz; 1:500) was used for detection. Slides were counterstained with DAPI.

### Statistical analysis

Data are presented as means ± SEM. Differences between groups were determined by student’s t test or ANOVA multiple comparisons as appropriate using in GraphPad Pro (GraphPad, San Diego, CA). Differences were considered to be significant at P < 0.05.

## Additional Information

**How to cite this article:** Wang, L. *et al*. Inhibition of epidermal growth factor receptor attenuates atherosclerosis via decreasing inflammation and oxidative stress. *Sci. Rep.* **7**, 45917; doi: 10.1038/srep45917 (2017).

**Publisher's note:** Springer Nature remains neutral with regard to jurisdictional claims in published maps and institutional affiliations.

## Supplementary Material

Supplementary Files
