# Supplementary material for: Inhibition of epidermal growth factor receptor attenuates atherosclerosis via decreasing inflammation and oxidative stress
Source: Sci Rep. 2017 Apr 4;7:45917. doi: 10.1038/srep45917 (PMC5379239; doi:10.1038/srep45917)
Supplement: Supplementary Files [file srep45917-s1.doc]

***Supplementary information***

**Inhibition of epidermal growth factor receptor attenuates atherosclerosis via decreasing inflammation and oxidative stress**

Lintao Wang^1,#^, Zhouqing Huang^2,#^, Weijian Huang^2^, Xuemei Chen^1^, Peiren Shan^2^, Peng Zhong^1^, Zia Khan^3^, Jingying Wang^1^, Qilu Fang^1^, Guang Liang^1^,* , Yi Wang^1^,*

*1 Chemical Biology Research Center, School of Pharmaceutical Sciences,* *Wenzhou Medical University, Wenzhou, Zhejiang,325035, China;*

*2 Department of Cardiology, the First Affiliated Hospital, Wenzhou Medical University, Wenzhou, Zhejiang,325035, China.*

*3 Department of Pathology and Laboratory Medicine, Western University, London, ON N6A5C1, Canada*

**Supplementary Figures and Legends**

Supplementary data including 20 figures and 1 table is available online.

**Table S1:** Primers used for real-time qPCR assay.

| Gene | Species | Primers(FW) | Primers(RW) |
| --- | --- | --- | --- |
| TGF-β | Mouse | TGACGTCACTGGAGTTGTACGG | GGTTCATGTCATGGATGGTGC |
| Collagen1 | Mouse | TGGCCTTGGAGGAAACTTTG | CTTGGAAACCTTGTGGACCAG |
| C-TGF | Mouse | ACTATGATGCGAGCCAACTGC | TGTCCGGATGCACTTTTTGC |
| TNF-α | Mouse | TGATCCGCGACGTGGAA | ACCGCCTGGAGTTCTGGAA |
| IL-6 | Mouse | GAGGATACCACTCCCAACAGACC | AAGTGCATCATCGTTGTTCATACA |
| ICAM-1 | Mouse | GCCTTGGTAGAGGTGACTGAG | GACCGGAGCTGAAAAGTTGTA |
| VCAM-1 | Mouse | TGCCGAGCTAAATTACACATTG | CCTTGTGGAGGGATGTACAGA |
| β-actin | Mouse | CCGTGAAAAGATGACCCAGA | TACGACCAGAGGCATACAG |
| MMP2 | Mouse | CAAGGACCGGTTTATTTGGC | ATTCCCTGCGAAGAACACAGC |
| Nox1 | Mouse | GGTTGGGGCTGAACATTTTTC | TCGACACACAGGAATCAGGAT |
| TNF-α | Human | CCCAGGGACCTCTCTCTAATC | ATGGGCTACAGGCTTGTCACT |
| IL-6 | Human | GCACTGGCAGAAAACAACCT | TCAAACTCCAAAAGACCAGTGA |
| β-actin | Human | CCTGGCACCCAGCACAAT | GCCGATCCACACGGAGTACT |

**Figure S1:** ApoE-/- mice were fed with HFD for 8 weeks, and treated with AG1478 (AG, 10mg/kg/day) or 542 (10 mg/kg/day) for 8 weeks by oral gavage. Serum levels of insulin were measured by Insulin Assay Kit (Nanjing Jiancheng, Jiangsu, China).. EGFR inhibitors failed to alter HFD-induced increased serum insulin level. (n=7 in each group; ^###^P<0.001, vs LFD; ns, not significant vs HFD).

**Figure S2: Quantification for staining results in Figure 1D.** (n=7 in each group, ^###^*P<*0.001, vs LFD group; ^**^*P*<0.01, vs HFD group)

**A B**


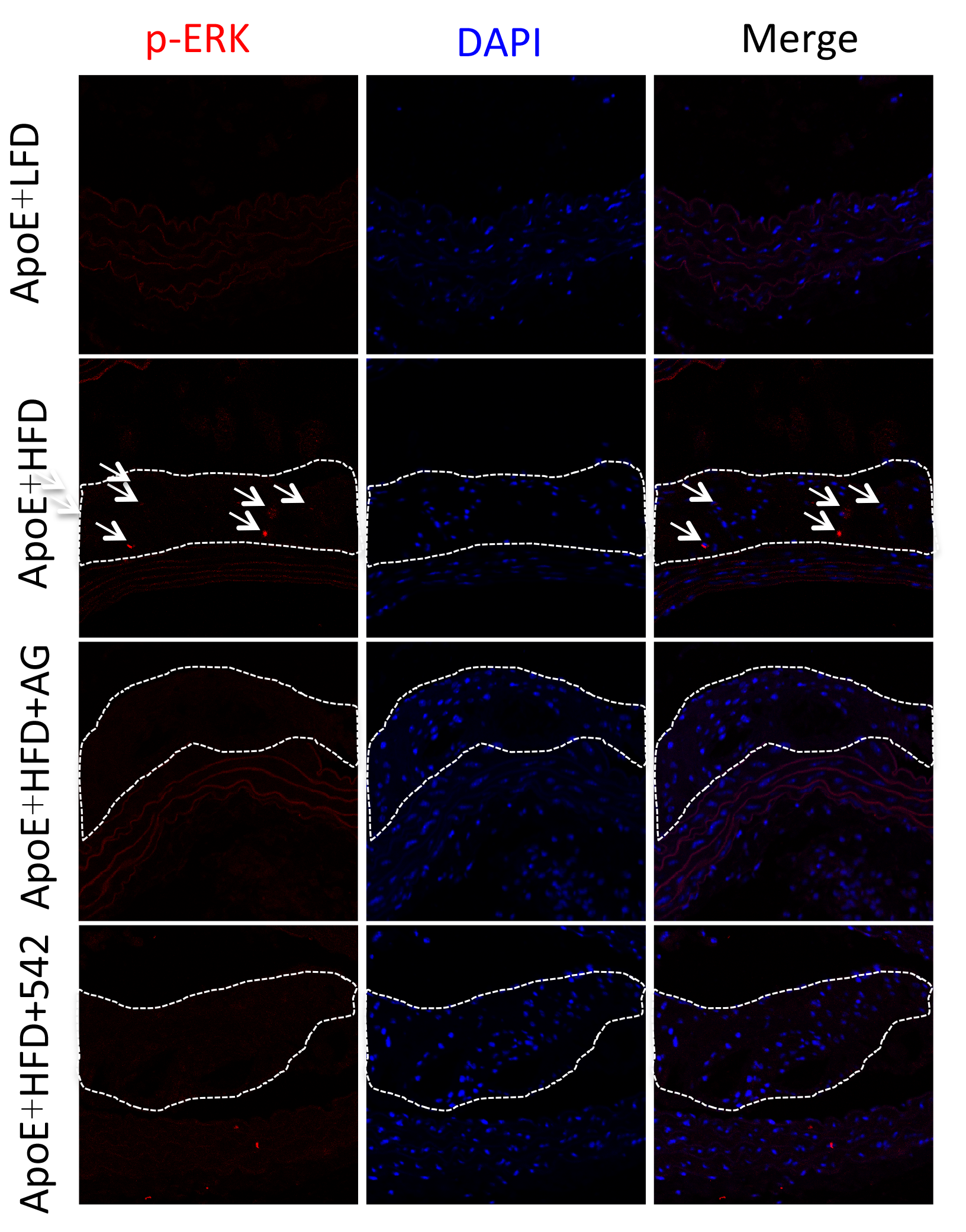

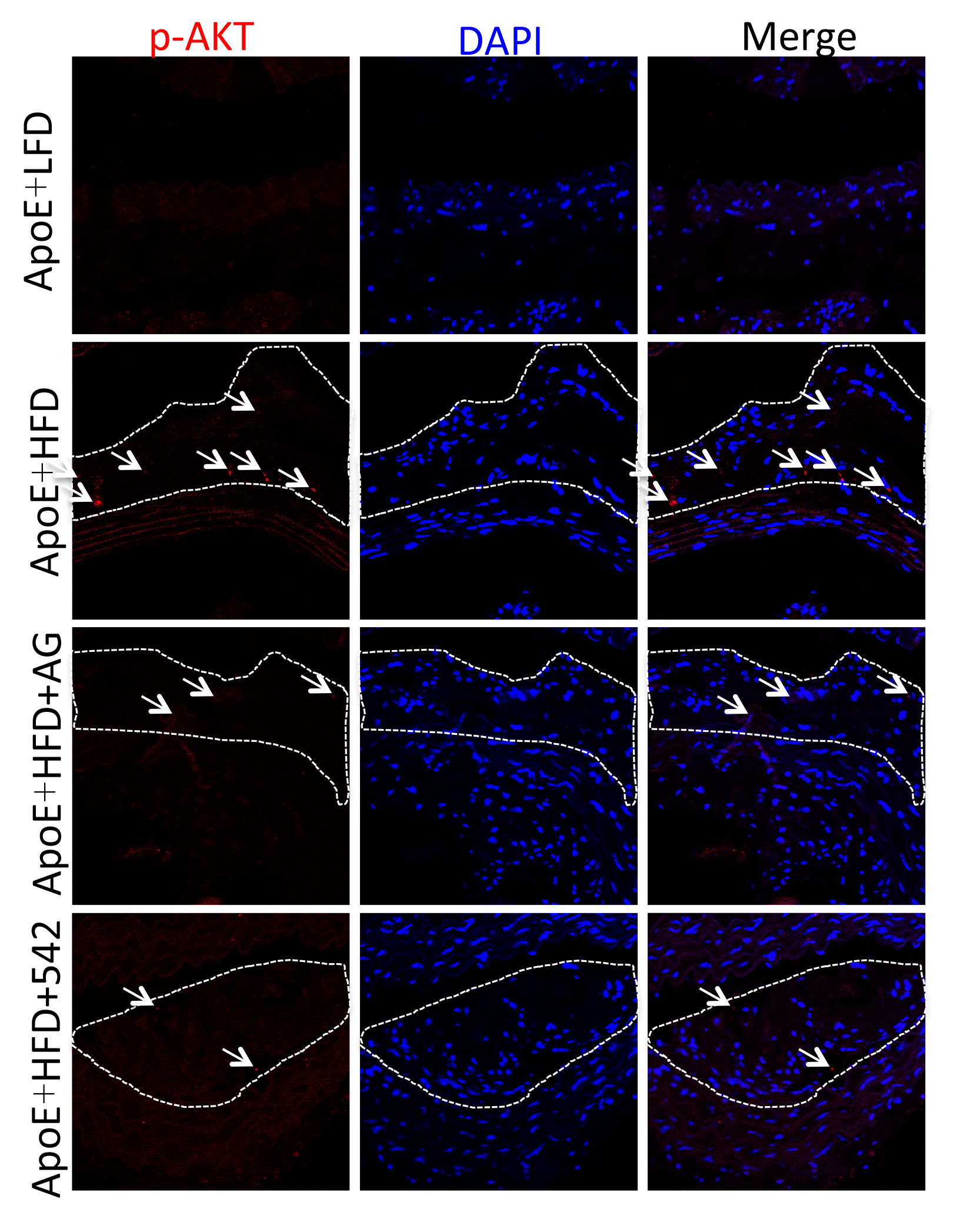


**C D**

**Figure S3:** A and B. Representative immunofluorescence staining for p-ERK (A) and p-AKT (D) in the aortic section. White arrows indicate p-ERK or p-AKT positive cells. Nuclei were stained with DAPI (blue). Antibodies against p-AKT were purchased from Cell Signaling (Danvers, MA, USA). Antibodies against p-ERK were purchased from Santa Cruz Biotechnology (Santa Cruz, CA). C and D. Quantification of the number of p-ERK or p-AKT positive cell in total cells (n=7 per group, ^###^*P*<0.001, vs the LFD group; ^***^*P*<0.001, vs the HFD group).

**A B**

**C**

**Figure S4:** Densitometric quantifications for Figure 1E. (n=7 per group, ^#^*P*<0.05, ^##^*P*<0.01, vs the LFD group; ^*^*P*<0.05, ^**^*P*<0.01, ^***^*P*<0.001, vs the HFD group).

**A**


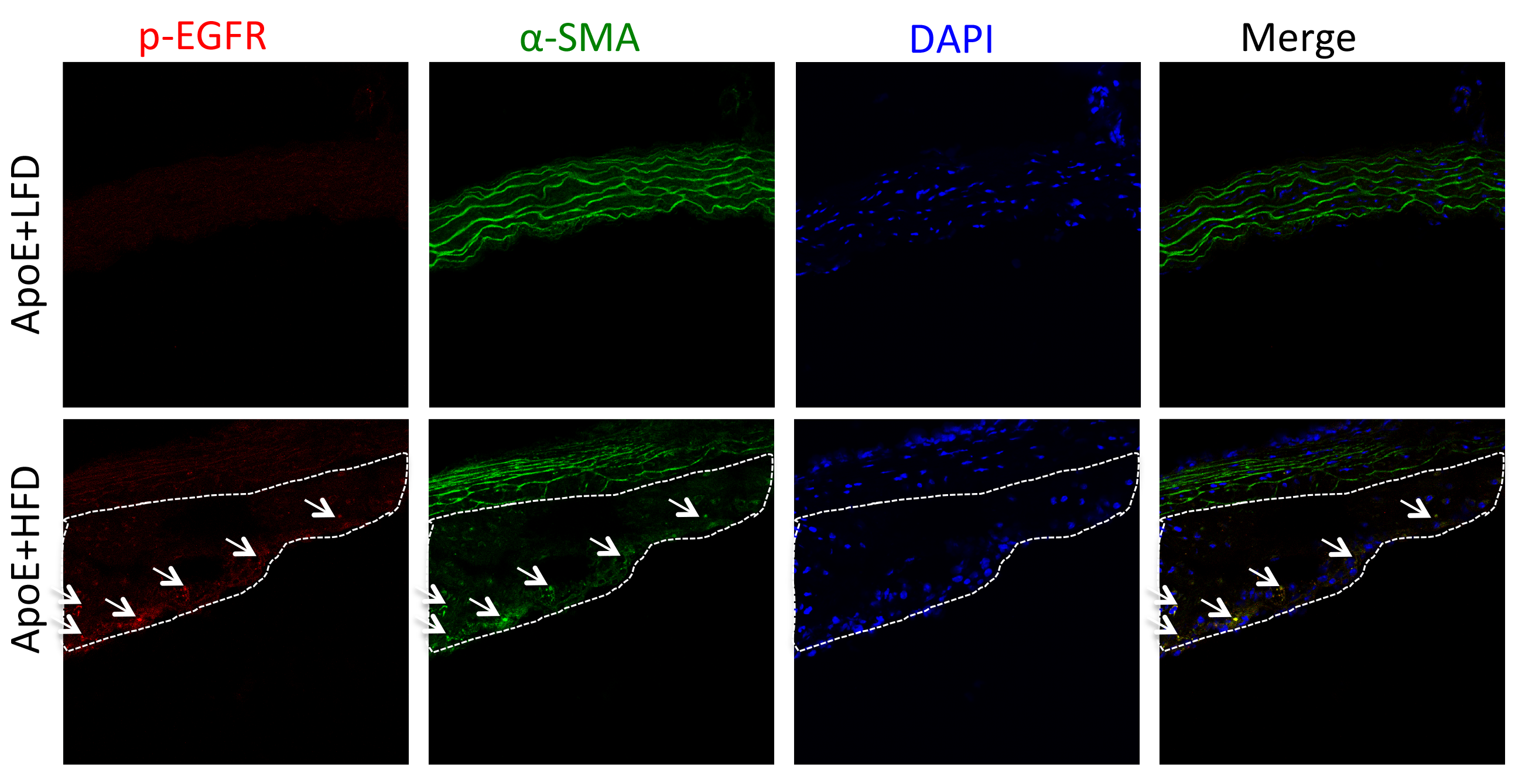


**B**


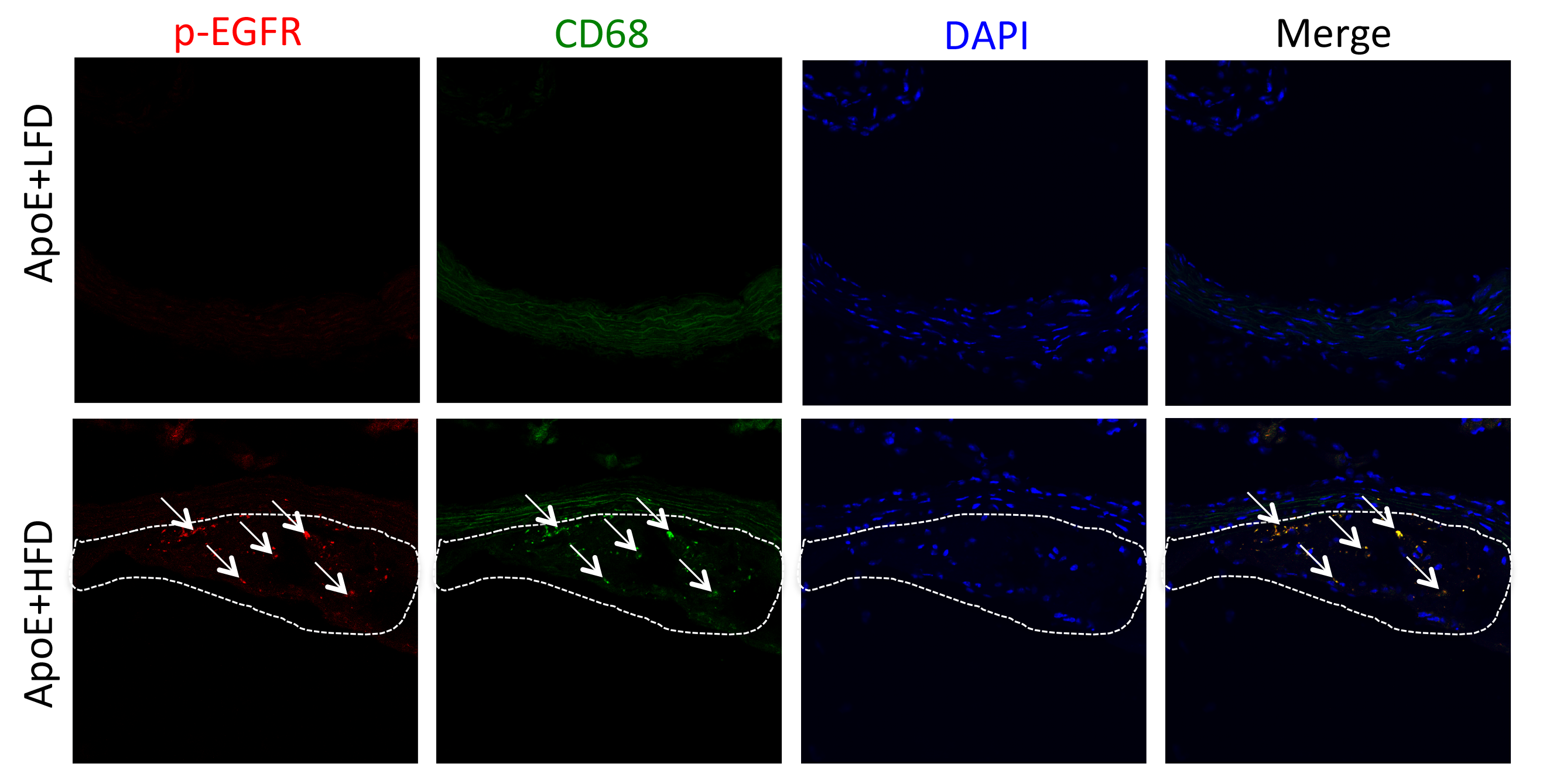


**C**


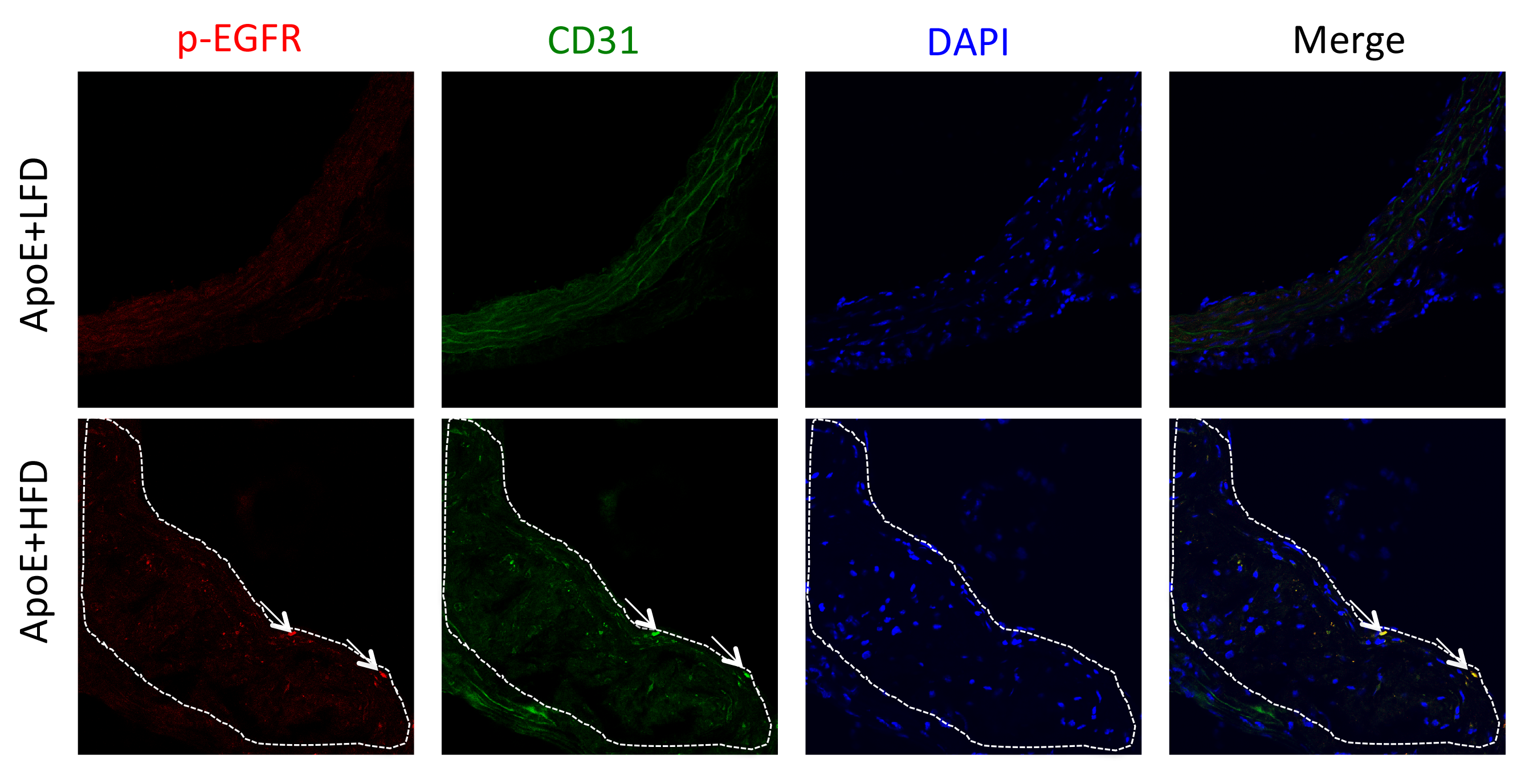


**D E F**

**Figure S5.** The p-EGFR localization at the atherosclerotic plaques in aortas of the ApoE-/- mice on LFD or HFD by colocalization immunofluorescence staining. (A) Representative immunofluorescence staining of p-EGFR and α-SMA; (B) Representative immunofluorescence staining of p-EGFR and CD68; (C) Representative immunofluorescence staining of p-EGFR and CD31. Nuclei were stained with DAPI (blue). (D-F) Quantification for panels A-C, respectively (n=7 per group, ^###^*P* <0.001, vs the LFD group). Antibody against p-EGFR (Rabbit polyclonal) was purchased from Cell Signaling (Danvers, MA, USA). Antibodies against CD68(Mouse monoclonal), smooth muscle α-actin (α-SMA, mouse monoclonal) and CD31(Mouse monoclonal) were purchased from abcam (Abcam, Cambridge, MA). FITC-conjugated secondary antibody (Anti-mouse) and TRITC-conjugated secondary antibody(Anti-rabbit) were purchased from abcam (Abcam, Cambridge, MA). DAPI was purchased from Beyotime (Beyotime Biotechnology, China).

**A B C**

**Figure S6:** Quantification for staining results in Figure 2D (A), 2E (B), and 2F (C). (n=7 per group, ^##^*P*<0.01, ^###^*P*<0.001, vs LFD group; ^*^*P<*0.05, ^**^*P*<0.01, ^***^*P*<0.001, vs HFD group)


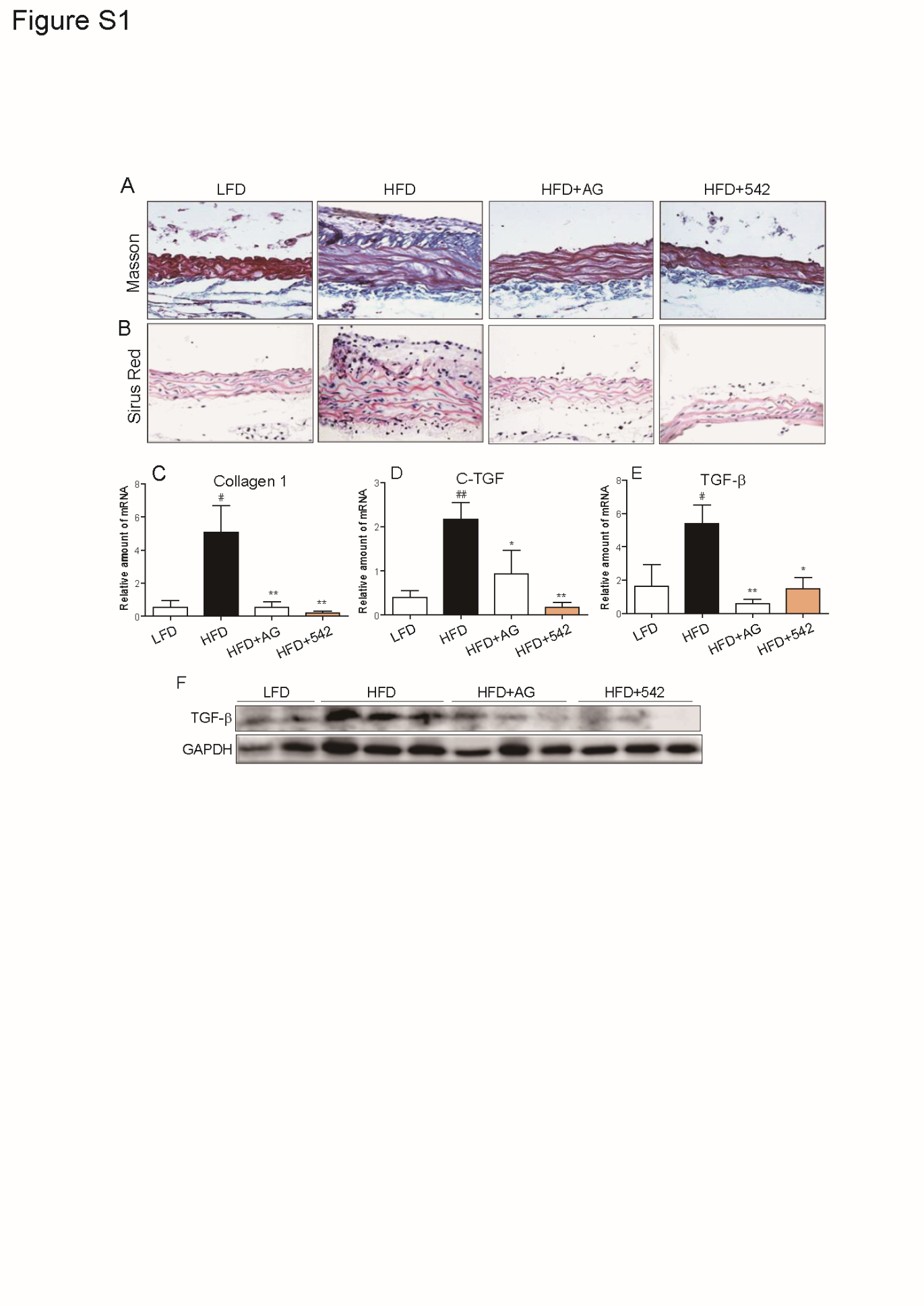


**Figure S7:** AG or 542 administration reduced fibrosis in the atherosclerosis plaque of HFD-fed ApoE^-/-^ mice. A-B. Representative images for Masson staining and Sirius red staining in the aortic section indicated EGFR inhibitors decreased collagen deposition and fibrosis. C-E. Real-time qPCR assay showed that AG and 542 reduced the mRNA production of pro-fibrosis genes including collagenⅠ(C), CTGF (D), and TGF-β (E) in the HFD mouse artery. F. Western blot analysis showed that AG and 542 reduced TGF-β expression in the HFD mouse artery. (n=7 per group, ^#^ *P<*0.05, ^##^*P*<0.01, vs the LFD group; ^*^*P*<0.05, ^**^*P*<0.01, vs the HFD group).

**A B**

**Figure S8:** Quantification for staining results shown in Figure 3G (A) and 3H (B). (n=7 per group, ^##^*P*<0.01, ^###^*P*<0.001, vs LFD group; ^*^*P<*0.05, ^**^*P<*0.01, ^***^*P<*0.001, vs HFD group)

**Figure S9:** Densitometric quantifications for Figure 4A. (n=4 independent experiments, ^*^*P*<0.05, vs control group; ns, not significant vs control group).

**Figure S10:** Quantification for staining results in Figure 4B. (n=4 independent experiments, ^###^*P*<0.001, vs control group; ^***^*P*<0.001, vs ox-LDL group).

.

**A B**

**Figure S11:** Densitometric quantifications for Figure 4C (A) and 4D (B). (n=4 independent experiments, ^###^*P*<0.001, vs control group; ^**^*P*<0.01, ^***^*P*<0.001, vs ox-LDL group).

**Figure S12:** Quantification for staining results in Figure 4E. (n=4 independent experiments, ^##^*P*<0.01, vs control group; ^**^*P*<0.01, ^***^*P*<0.001, vs ox-LDL group).


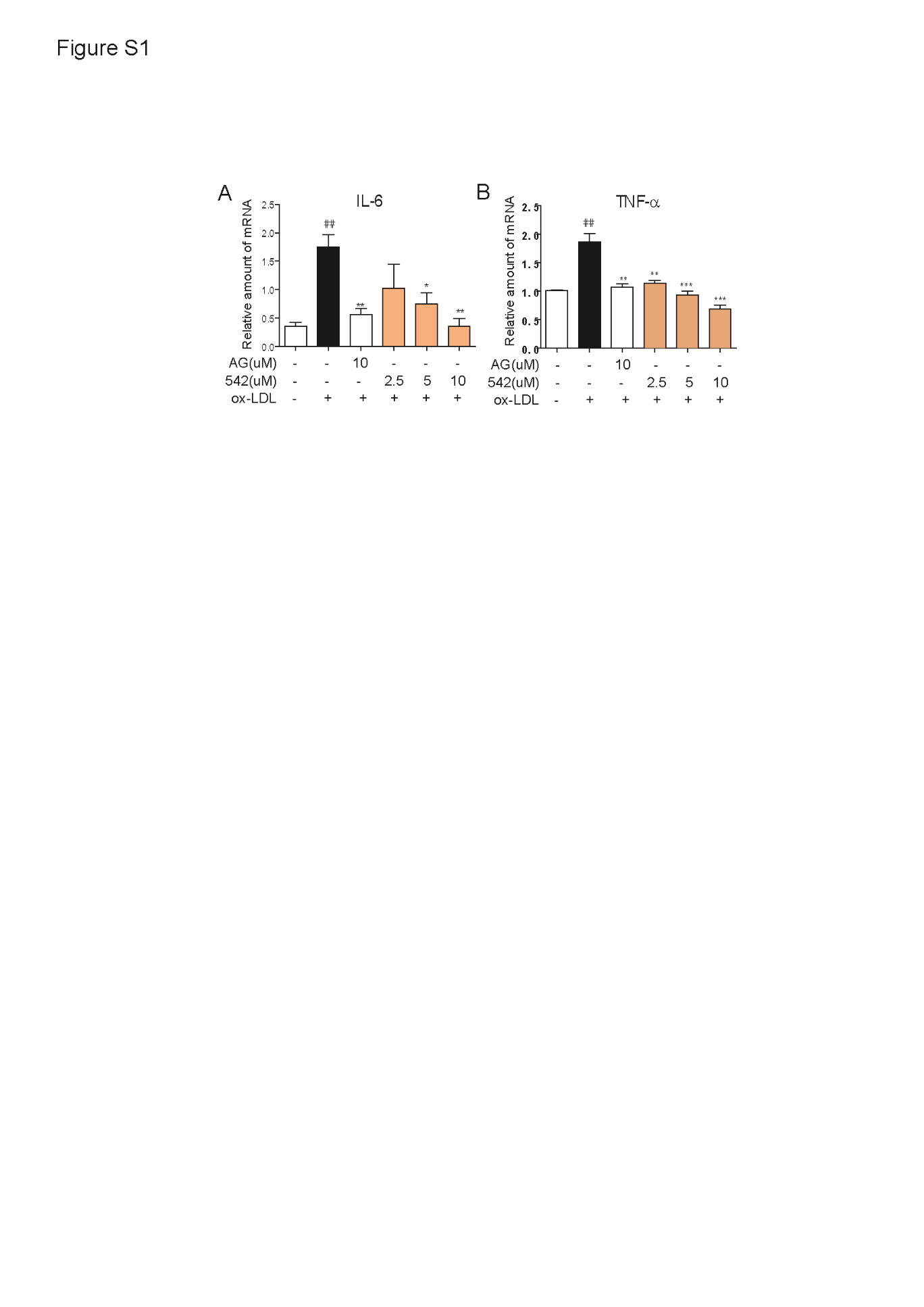


**Figure S13:** AG and 542 block oxLDL-induced cytokine expression in macrophages. Primary macrophages were pretreated with 542 (2.5, 5 or 10 μM), AG1478 (10 μM), or vehicle (DMSO, 1 μL) for 1 h and then stimulated with ox-LDL (50 μg/mL) for 6h. Total mRNAs were extracted from the cell lysates and the mRNA levels of IL-6 (A) and TNF-α (B) were detected by real-time qPCR analysis. (n=4 independent experiments, ^##^*P<*0.01, vs control group; ^*^*P*<0.05, ^**^*P*<0.01, ^***^*P*<0.001, vs ox-LDL group).


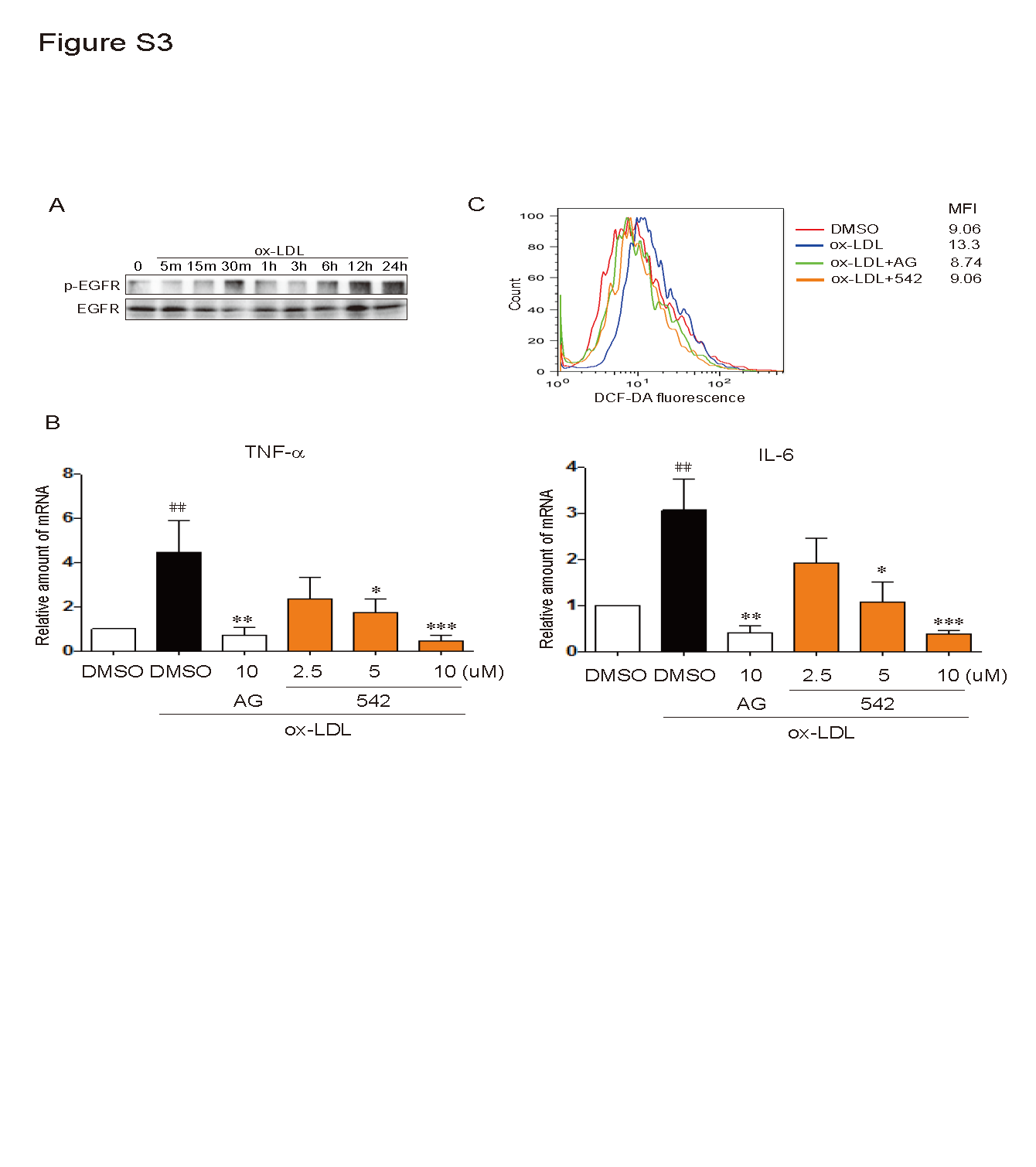


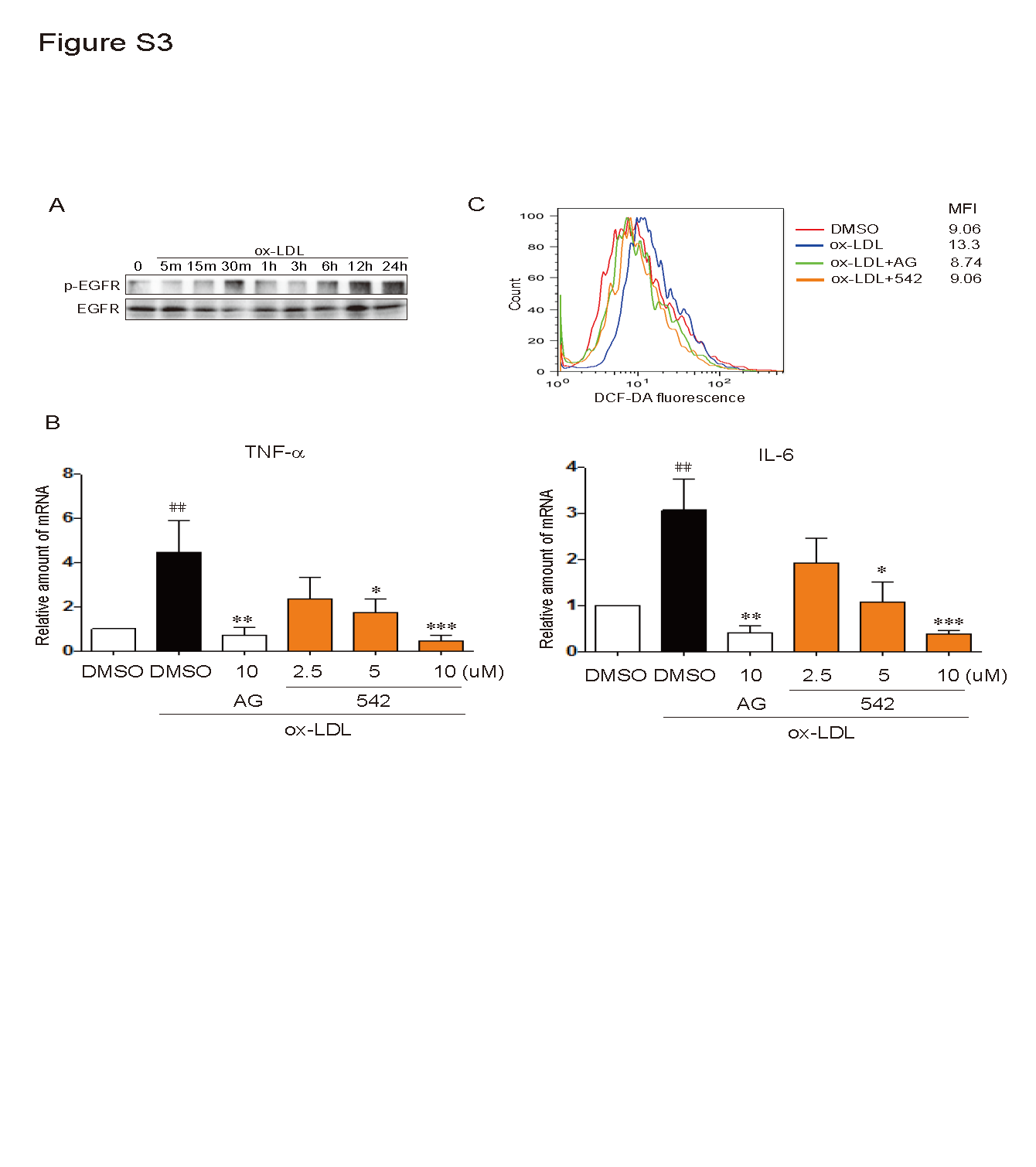


**Figure S14.** AG and 542 block oxLDL-induced cytokine expression in human vascular smooth muscle cells (VSMCs). A. oxLDL activate EGFR in VSMCs. VSMCs were stimulated with ox-LDL(50 μg/ml) for different time points. The cell lysates were harvested and proceeded to western blot analysis for p-EGFR/EGFR. B. AG and 542 block oxLDL-induced cytokine expression in VSMCs. VSMCs were pretreated with 542 (2.5, 5 or 10 μM), AG1478 (10 μM), or vehicle (DMSO, 1 μL) for 1 h and then stimulated with ox-LDL (50ug/ml) for 6h. Total mRNAs were extracted from the cell lysates and the mRNA levels of IL-6 (A) and TNF-α (B) were detected by real-time qPCR analysis. (n=4 independent experiments, ^##^*P<*0.01, vs control group; ^*^*P*<0.05, ^**^*P*<0.01, ^***^*P*<0.001, vs ox-LDL group).

**A B**

**Figure S15:** Quantification for staining results shown in Figure 5A. (n=4 independent experiments, ^##^*P*<0.01, ^###^*P*<0.001, vs control group; ^**^*P*<0.01, ^***^*P*<0.001, vs ox-LDL group).


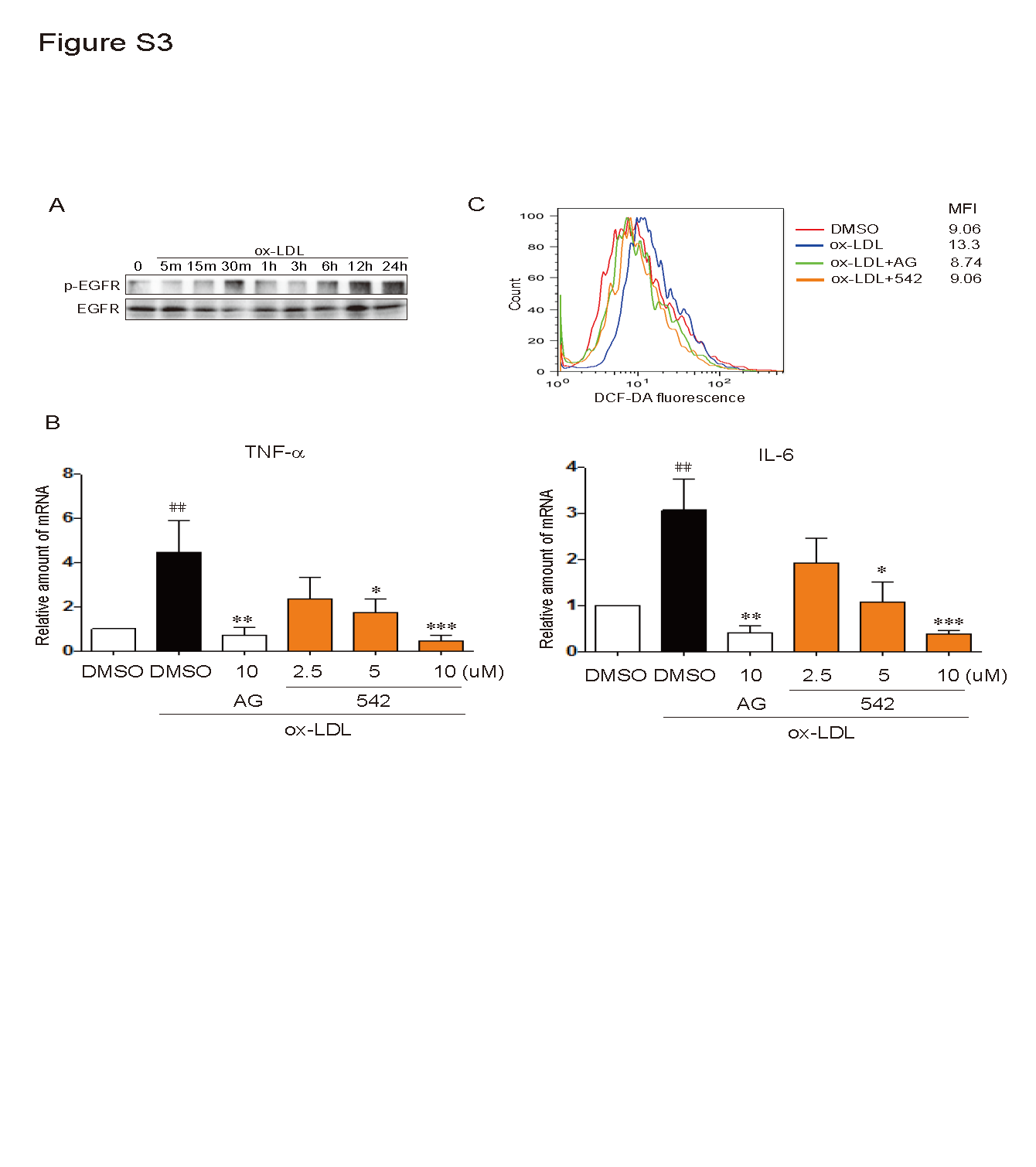


**Figure S16.** AG and 542 block oxLDL-induced ROS production in human vascular smooth muscle cells (VSMCs). AG and 542 inhibited the production of H_2_O_2_ level induced by ox-LDL. VSMCs were pretreated with 542 and AG at 10 μM for 1 h, followed by the incubation with ox-LDL (50 μg/mL) for 30min. DCFH-DA probes were loaded and cells were processed to flow cytometry analysis for H_2_O_2_ level. (n=4 independent experiments).


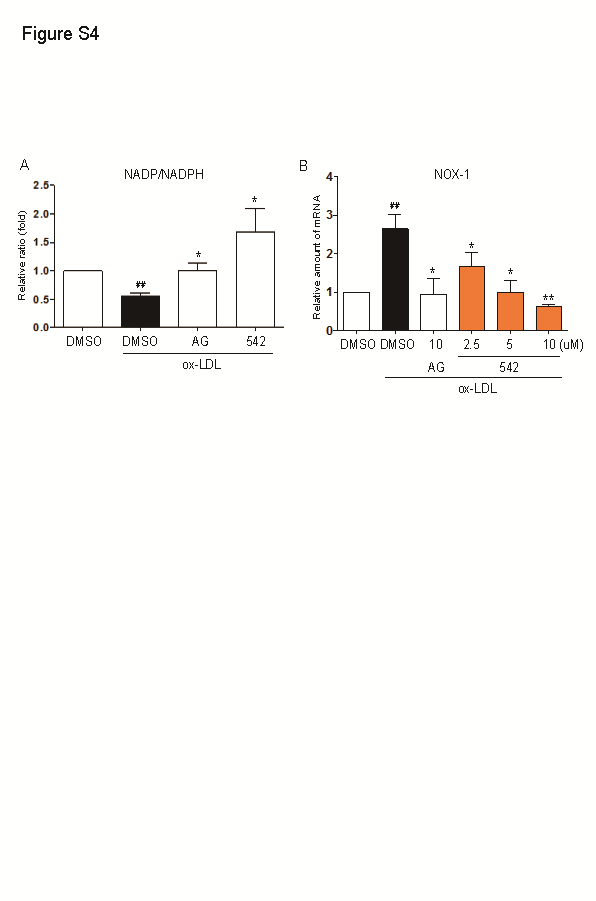


**Figure S17. AG and 542 block oxLDL-induced NOX-1 expression and activation in macrophages.** A. AG and 542 block oxLDL-induced NOX-1 expression. Primary macrophageds were pretreated with 542 (10 μM), AG1478 (10μM), or vehicle (DMSO, 1 μL) for 1 h and then stimulated with ox-LDL (50ug/ml) for 6h. NOX activity in cells was tested using NADP/NADPH Quantification colorimetric Kit. B. AG and 542 block oxLDL-induced NOX-1 expression. Primary macrophageds were pretreated with 542 (2.5, 5 or 10 μM), AG1478 (10μM), or vehicle (DMSO, 1 μL) for 1 h and then stimulated with ox-LDL (50ug/ml) for 6h. Total mRNAs were extracted from the cell lysates and the mRNA level of NOX-1 were detected by real-time qPCR analysis. (n=4 independent experiments, ^¥¥^*P*<0.01, vs control group; ^*^*P*<0.05, ^**^*P*<0.01, vs ox-LDL group).


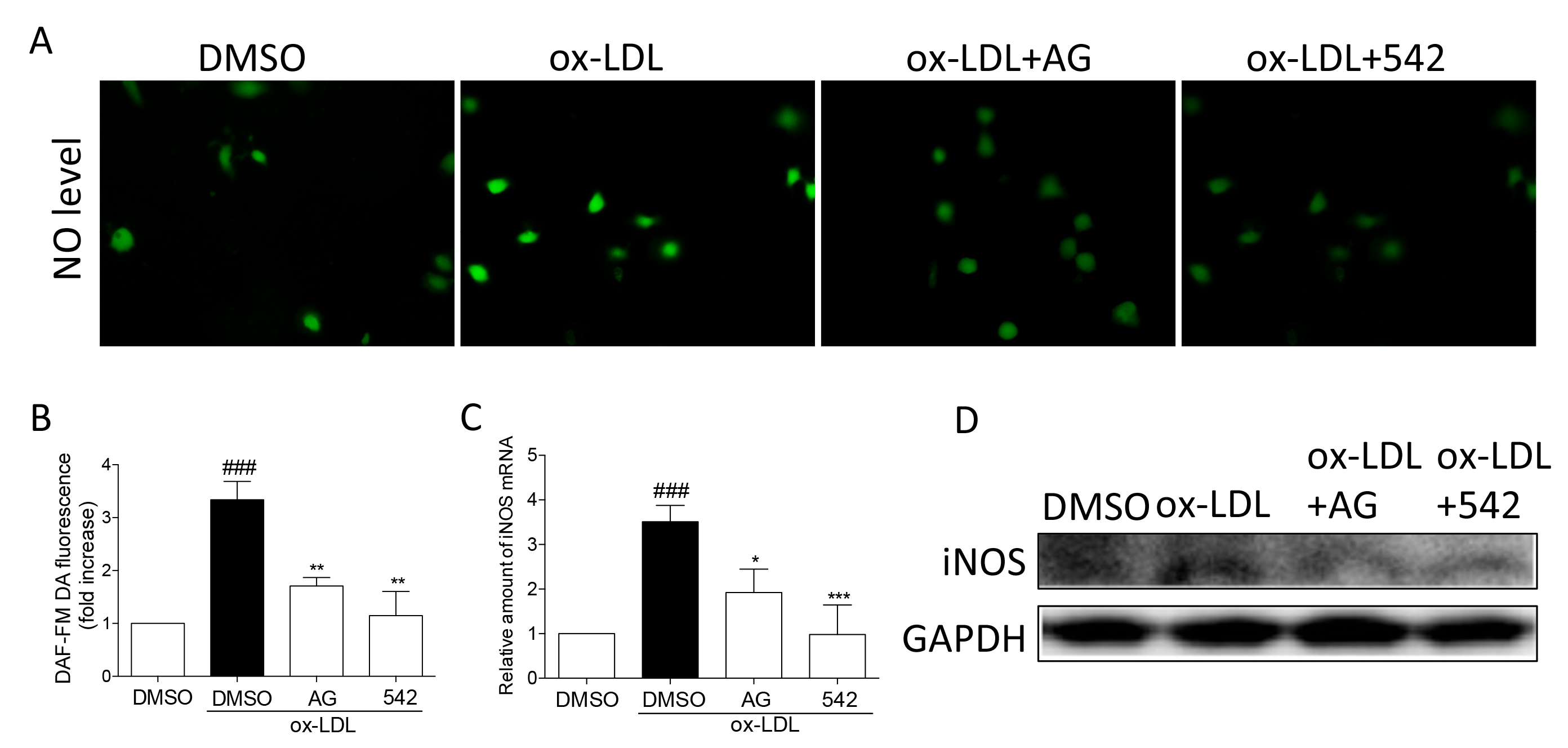


**Figure S18:** AG and 542 inhibits oxLDL-induced NO generation in macrophages. A. Primary macrophages that were pretreated with 542 (10 μM), AG1478 (10μM), or vehicle (DMSO, 1 μL) for 1 h were incubated with ox-LDL (50 µg/mL) for 6h. DAF-FM DA probes for NO were loaded and the NO positive cells were detected using fluorescence microscope (Nikon). B. Quantification for panel A. C. Primary macrophages were pretreated with 542 (10 μM), AG1478 (10μM), or vehicle (DMSO, 1 μL) for 1 h and then stimulated with ox-LDL (50ug/ml) for 6h. Total mRNAs were extracted from the cell lysates and the mRNA level of iNOS was detected by real-time qPCR analysis. D. Primary macrophageds were pretreated with 542 (10 μM), AG1478 (10μM), or vehicle (DMSO, 1 μL) for 1 h and then stimulated with ox-LDL (50ug/ml) for 12h. Total proteins were extracted to detect the levels of iNOS using western blot analysis. (n=3 independent experiments, ^###^*P*<0.001, vs control group; ^*^*P*<0.05, ^**^*P*<0.01, ^***^*P*<0.001, vs ox-LDL group). [DAF-FM DA was purchased from Beyotime (Beyotime Biotechnology, China); Antibody against iNOS was purchased from Cell Signaling (Danvers, MA, USA); iNOS primer: FW: CAGCTGGGCTGTACAAACCTT, RW: CATTGGAAGTGAAGCGTTTCG]

**A B**

**Figure S19:** Quantification for staining results shown in Figure 5E (A) and 5F (B). (n=4 independent experiments, ^###^*P*<0.001, vs control group; ^***^*P*<0.001, vs ox-LDL group).

**Figure 20**. Gels/blots with the cropping lines.
